# Stable Radical Isoporphyrin Copolymer Prepared with Di(phenylphosphane)

**DOI:** 10.3390/molecules29133056

**Published:** 2024-06-27

**Authors:** Yiming Liang, Antoine Bonnefont, Vasilica Badets, Corinne Boudon, Michel Goldmann, Guillaume Diot, Sylvie Choua, Nolwenn Le Breton, Laurent Ruhlmann

**Affiliations:** 1 Zhengzhou Research Base, National Key Laboratory of Cotton Bio-Breeding and Integrated Utilization, School of Agricultural Sciences, Zhengzhou University, Zhengzhou 450001, China; liangyiming112@163.com; 2 Institut de Chimie, UMR CNRS 7177, Université de Strasbourg, 4 Rue Blaise Pascal, CS 90032, F-67081 Strasbourg Cedex, France; pierre-antoine.bonnefont@grenoble-inp.fr (A.B.); badets@unistra.fr (V.B.); cboudon@unistra.fr (C.B.); sylvie.choua@unistra.fr (S.C.); nlebreton@unistra.fr (N.L.B.); 3 LEPMI, Université 589 Grenoble Alpes, Université Savoie Mont Blanc, CNRS, 590 Grenoble INP, F-38000 Grenoble, France; 4 Institut des Nanosciences de Paris, UMR CNRS 7588, Sorbonne Université, 4 Place Jussieu, Boîte Courrier 840, F-75252 Paris, France; michel.goldmann@insp.jussieu.fr (M.G.); guillaumegilleswillydiot@gmail.com (G.D.); 5 UFR des Sciences Fondamentales et Biomédicales, Université Paris-Cité, 45 Rue des Saints Pères, F-75006 Paris, France

**Keywords:** porphyrin, phosphonium, EPR spectroscopy, EQCM, electrochemistry, photocurrent

## Abstract

Two diphosphanes with variable-length ligands tested as nucleophiles to prepare isoporphyrin copolymers in the presence of ditolylporphyrin of zinc (ZnT_2_P) prevented the oxidation of the diphosphine ligand. This paper demonstrates the power of this approach and describes the photoelectrocatalytic properties. The obtained copolymers were characterized by UV–vis spectroscopy, X-ray photoelectron spectroscopy, atomic force micrograph (AFM), EQCM (Electrochemical Quartz Cristal Microbalance) and electrochemistry. Their impedance properties (EIS) were studied and their photovoltaic performances were also investigated by photocurrent transient measurements under visible light irradiation.

## 1. Introduction

Over the years, several extended porphyrin structures have been prepared and studied with a particular interest in the dependence of the geometric factors on their physical properties. Structural flexibility is determined by the nature of the bridge. To observe efficient interactions between the different chromophores, a large variety of bridging chains have been used as spacers in the building of dimers, but only a few papers were devoted to phosphanylporphyrins. Electrochemistry has been shown to be an appropriate synthetic tool in the coupling of porphyrins.

Phosphorus ligands are useful nucleophiles and could nucleophilic attack the π cation radicals of metalloporphyrins.

In an original study, Smith and colleagues demonstrated that triphenylphosphine (PPh_3_) functions as a nucleophile in the meso position when reacting with the cation radical of the Zn(II) octaethylporphyrin complex (ZnOEP), initially formed via chemical oxidation [1,2]. Additionally, Shine et al. described the reactivity at the *meso*-position of the zinc(II) tetraphenylporphyrin cation radical ZnTPP^+●^, which was also chemically generated, in the presence of PPh_3_ [3,4]. Subsequently, Latos-Grażyński et al. reported the chemical *β*-functionalization of preformed Fe^III^TPP(ClO_4_)_2_ with PPh_3_, resulting in the formation of the dimer [Fe^III^TPP-*β*-^+^PPh_3_]_2_O(ClO_4_), albeit with modest yields in both *meso*- and *β*-phosphonium-based porphyrins [5]. Giraudeau et al. made significant strides in improving the yield of phosphonium-based porphyrins by continuously generating the porphyrin π-cation radical in the presence of PPh_3_ through controlled potential electrolysis. Under these conditions, Zn^II^TPP-*β*-^+^PPh_3_ClO_4_^−^ was produced with a 50% yield [6].

Lastly, the electrochemical oxidation of zinc 5,15-*bis*(*p*-tolyl)-10-phenylporphyrin in the presence of PPh_3_ resulted in the formation of *meso*-substituted triphenylphosphonium porphyrin, coordinated by one O=PPh_3_ molecule, with a good yield of 84% [7].

In a direct one-pot electrochemical reaction, the bis(diphenylphosphino)acetylene ligand acts as a nucleophile allowing the electrosynthesis of diporphyrins with a diphosphonium spacer bridged directly at their meso position upon oxidation of the zinc *meso*-tetraphenyl porphyrin (ZnTPP) at an applied potential of 0.80 V *vs.* SCE [8]. We also described the efficient synthesis of various diphosphonium-bridged homometallated porphyrin dimers and trimers by controlled potential electrolysis in a direct one-pot electrochemical reaction using more or less rigid diphosphanes with variable length [9,10]. It clearly illustrates that phosphorus ligands act as powerful nucleophiles which allow electrochemical *β*-substitutions on *meso*-tetra-phenylporphyrins. However, such diphosphane compounds are easily oxidized which can limit the use of such ligands for the electrosynthesis, which is conditioned by the applied potential as well as the potential of the first oxidation of the porphyrin.

It must be noted that not only diphosphonium ligands can act as bridges between porphyrins. In the case of an aminophenyl derivative with –NH_2_ groups at the para position of the phenyl substituents, for example in 5,10,15,20-tetrakis(4-amino-phenyl)porphyrin or 5,15-bis(4-aminophenyl)-10,20-bis(4-carbomethoxyphenyl)porphyrin, oxydation gives a highly interconnected nanofibrous network and a linear porphyrin polymer [11,12,13]. The mechanism is considered analogous to that found in aniline polymerization, except that the attack of the electrophilic nitrogen must occur exclusively to the *meta*-positions of the aminophenyl substituents of another porphyrin molecule. Furthermore, oxidative electrodeposition of CoTSPP (tetrakis 4-sulfonatophenyl Co porphyrin) on ITO electrode from NaOH 0.1 M aqueous solution has been also studied [14], following the procedure previously described by Bedioui et al. for Ni-porphyrins electrodeposition [15].

**Scheme 1:**
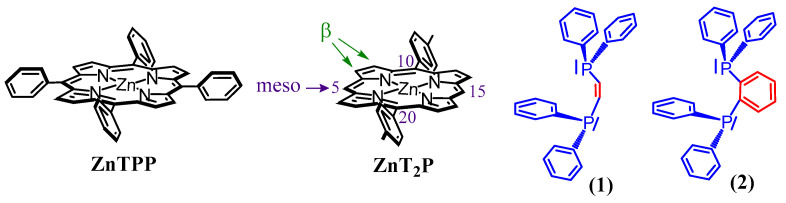
Representation of the zinc-tetraphenylporphyrin (**ZnTPP**), zinc-*meso*-5,15-ditolyl-porphyrin (**ZnT_2_P**), *cis*-1,2-bis(diphenylphosphino)ethene (**1**), and 1,2-bis(diphenylphosphino)benzene (**2**).

Recently, we also discovered the formation of stable isoporphyrins copolymers linked by pyridyl ligands [16,17]. Our group has shown that stable isoporphyrins copolymers could be prepared during porphyrin electro-oxidation when sterically less hindered porphyrin is used, such as the zinc-*meso*-5,15-ditolyl-porphyrin (**ZnT_2_P**), which presents only two meso positions occupied by one substitutable proton at positions C10 and C20 (Scheme 1).

**Scheme 2:**
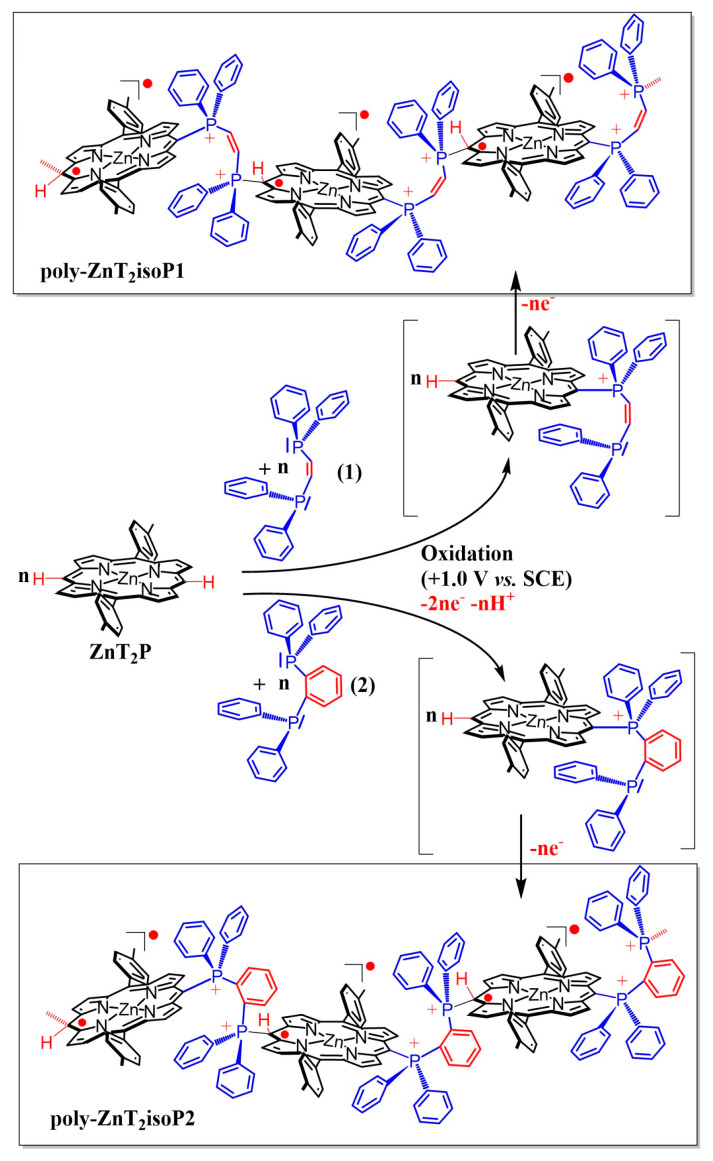
Representation of electropolymerization reaction from zinc-*meso*-5,15-ditolyl-porphyrin (ZnT_2_P), *cis*-1,2-bis-(diphenyl-phosphino)ethene (**1**), and 1,2-bis-(diphenylphosphino)-benzene (**2**).

In this respect, we report hereafter, the formation of stable isoporphyrin copolymers (Scheme 2) from the ditolyl porphyrin of zinc, **ZnT_2_P** in the presence of diphosphanes such as *cis*-1,2-bis(diphenylphosphino)ethene (**1**), and 1,2-bis(diphenylphosphino)benzene (**2**) (Scheme 1). The morphology of the copolymers has been studied by atomic force micrograph (AFM). The obtained copolymers were characterized by UV–vis spectroscopy, X-ray photoelectron spectroscopy, EQCM (Electrochemical Quartz Cristal Microbalance) and electrochemistry. Their impedance properties (EIS) were studied and their photovoltaic performances were also investigated by photocurrent transient measurements under visible light irradiation.

## 2. Results and Discussion

### 2.1. Electropolymerization of Isoporphyrin Copolymers

As already reported, in the presence of various nucleophiles, the second oxidation step of the tetraphenyl porphyrin of zinc (ZnTPP) became irreversible and a new cathodic peak appeared, as observed in the reduction of electrochemically generated isoporphyrins [18,19,20,21,22].

**Figure 1:**
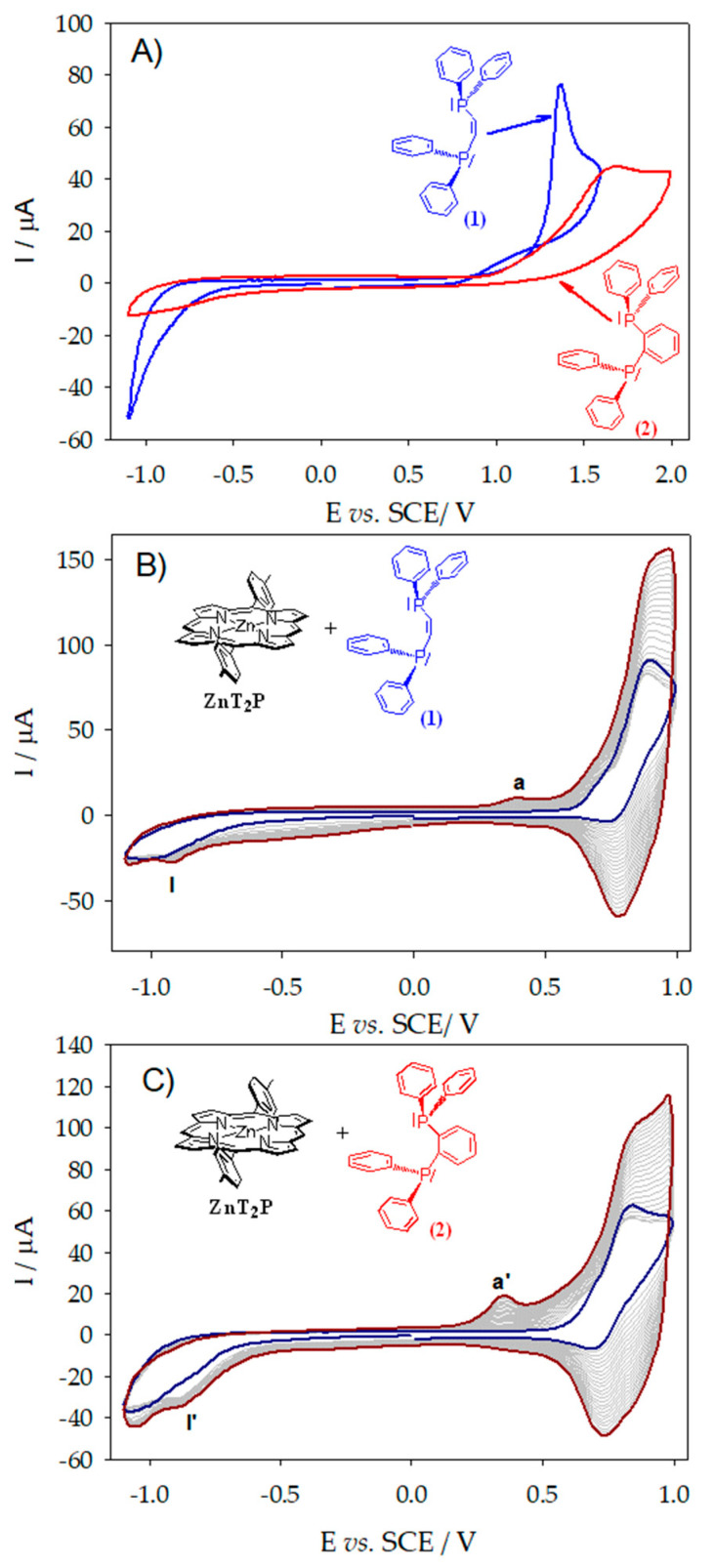
(**A**) Cyclic voltammograms recorded of cis-1,2-bis(diphenylphosphino)ethene and 1,2-bis(diphenylphosphino)benzene in CH_3_CN/1,2-C_2_H_4_Cl_2_ (3/7) with 0.1 mmol·L^−1^ NBu_4_PF_6_, v = 0.1 V·s^−1^. Cyclic voltammograms recorded during 25 iterative scans conducted between −1.1 V and +1.0 V *vs.* SCE in the presence of **ZnT_2_P** (0.25 mmol·L^−1^) with (**B**) *cis*-1,2-bis(diphenylphosphino)ethene (0.25 mmol·L^−1^) and (**C**) 1,2-bis(diphenylphosphino)benzene (0.25 mmol·L^−1^) in a 1,2-C_2_H_4_Cl_2_/CH_3_CN (7/3) solution with NBu_4_PF_6_ (0.1 mol·L^−1^). WE: ITO. S = 1 cm^2^. v = 0.1 V·s^−1^. Blue curve: first scan (*n* = 1). Red curve: final scan (*n* = 25).

In the case of the applied potential close to the second oxidation of the ZnTPP porphyrin (1.16 V *vs.* SCE) in the presence of tertiary diphosphane *cis*-1,2-bis(diphenylphosphino)ethene (**1**) or 1,2-bis(diphenylphosphino)benzene (**2**), oxidation of the di(phenylphosphane) occurs generating first the diphosphine cation radical, which is fully converted to the diphenylphosphino monooxide without formation of the dimer of porphyrin. The only product formed after purification was the diphenylphosphino monooxide. If using ZnTPP, no copolymer is obtained applying the potential at the first oxidation of the ZnTPP (0.80 V *vs.* SCE) where only the dimer of porphyrin with diphosphonium spacer is obtained as already published [8]. It must be noted that at this potential first oxidation of the porphyrin, the oxidation of the diphosphane ligand does not occur. Indeed, irreversible oxidation waves have been measured for *cis*-1,2-bis(diphenylphosphino)ethene (**1**) and 1,2-bis(diphenylphosphino)benzene (**2**) at +1.37 V and +1.68 V *vs.* SCE (Table 1 and Figure 1A) corresponding to the oxidation of the -P(Ph)_2_- phosphane group. This result is probably due to the steric hindrance of the ZnTPP porphyrin which leads to the slower kinetic of the nucleophilic attack at the meso positions (Scheme 1) [11,12].

In the case of the zinc-*meso*-5,15-ditolyl-porphyrin (**ZnT_2_P**), two sides occupied by hydrogen at the meso position 5 and 15 and at *β* positions are present (Scheme 1), leading to the decrease in the steric hindrance with faster nucleophilic attack at meso positions 5 and 15. Thus, oxidation at the first oxidation of **ZnT_2_P** should be more favorable to generate copolymers of isoporphyrin [16].

Cyclic voltammograms during electropolymerization of **ZnT_2_P** in the presence of *cis*-1,2-bis(diphenylphosphino)ethene (**1**) or 1,2-bis(diphenylphosphino)benzene (**2**) conducted between −1.1 V and +1.0 V *vs.* SCE are shown in Figure 1B,C.

**Figure 2:**
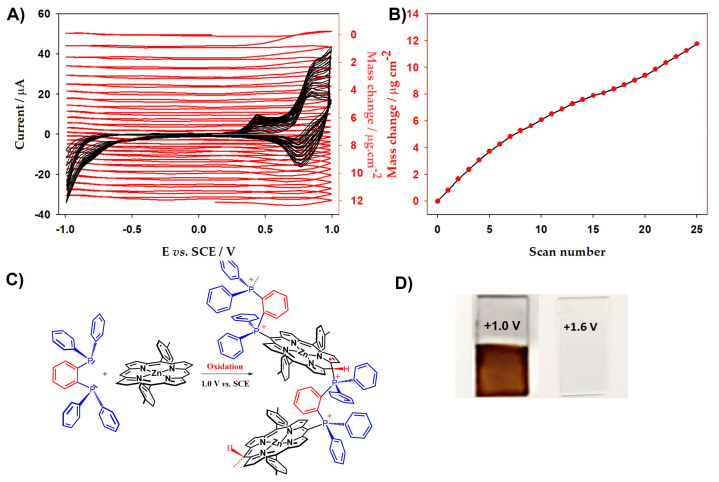
(**A**) Electrochemical quartz crystal microbalance measurements (Δ*m*) and consecutive cyclic voltammograms (first 25 scans) of **poly-ZnT_2_isoP2** and 1,2-bis(diphenylphosphino)benzene ligand. (**B**) Mass change (Δ*m*) of the first 25 scans calculated from Sauerbrey’s equation versus the number of scan n of **poly-ZnT_2_isoP2**. (**C**) Proposed redox reactivity of **poly-ZnT_2_isoP2**. (**D**) Figure of **poly-ZnT_2_isoP2** copolymer films deposited at 25 scans between −1.0 V and 1.0 V (v = 100 mV·s^−1^) on the ITO and after oxidized to 1.6 V on the ITO.

Upon the increase in the iterative scans n, the oxidation waves continue to increase, showing the growth of copolymer materials (Figure 1B,C). Finally, brown deposited films could be observed on the ITO surface (Figure 2D) showing the electrodeposition of the film.

The electrochemical synthesis of the polyisoporphyrin copolymers relies on the previously reported E_ox1_C_N1_E_ox2_C_B1_(E_ox3+n_C_B1+n_)_n_ process of nucleophilic substitution on porphyrins [16]. The mechanism of electropolymerization can be explained as follows: First, the porphyrin radical cation (ZnT_2_P^+•^, electrochemical step E_ox1_) is electrogenerated at 0.79 V *vs.* SCE. Then, ZnT_2_P^+•^ can be attacked by a diphenylphosphane groups (chemical step C_Nmeso_) at *meso*-carbon position to yield an isoporphyrin. This later compound can be oxidized (electrochemical step E_ox2_) and the hydrogen atom initially located on the *meso*-carbon is released inducing the rearomatization of the porphyrin (chemical step C_B1_) which give the mono-substituted porphyrin ZnT_2_P-*meso*-(Ph)_2_P^+^-Ph-P(Ph)_2_ PF_6_^−^. At this stage, we have already obtained the monosubstituted porphyrin with one phosphonium covalently connected to the porphyrin and one pendant phosphane, which is still active for nucleophilic attack. Thus, the oxidation of the monosubstituted porphyrin will generate the *β* radical cation porphyrin (E_ox3_ step) which can react itself (nucleophilic attack of the pendant phosphane group –P(Ph_2_)), forming isoporphyrin radical copolymer (C_N2_ step) with diphosphonium spacers (Scheme 2 and Supplementary Materials Schemes S1 and S2). Thus, the first oxidation of the ZnT_2_P generated the *β*-radical cation porphyrin ZnT_2_P^+●^, the di(phenylphosphane) ligand could react with ZnT_2_P^+●^ to form stable poly-isoporphyrin radical copolymers. Indeed, isoporphyrin radical films were found to be highly stable in the presence of dioxygen (still stable in the solid state even after four years).

### 2.2. Electrochemical Quartz Cristal Microbalance (EQCM)

The formation of **poly-ZnT_2_isoP1** and **poly-ZnT_2_isoP2** prepared from **ZnT_2_P** and *cis*-1,2-bis(diphenylphosphino)ethene) (**1**) or 1,2-bis(diphenylphosphino)benzene) (**2**) has been monitored in situ by EQCM with iterative scans between −1.0 V and +1.0 V *vs.* SCE (Figure 2 and Figure S1).

Electropolymerization occurs in the presence of diphosphane ligand and **ZnT_2_P** via the oxidation. Further, the trace of the first scan in Figure 2A shows a significant decrease in the resonance frequency and thus an increase in the deposited mass at the first oxidation of the porphyrin, i.e., electropolymerization occurs upon the formation of the radical cation porphyrin ZnT_2_P^+●^ in the presence of 1,2-bis(diphenylphosphino)benzene (**2**).

The resonance frequency displays a significant decrease meaning an increase in the deposited mass during the electropolymerization as shown in Figure 2B. Indeed, according to the Sauerbrey’s equation, the decrease in resonance frequency in Figure 2 corresponds to the increase in deposited mass. The mass of the copolymer film increased with the increase in number of potential cycle n (Figure 2B). Similar results have been obtained in the case of **poly-ZnT_2_isoP1** (Figure S1). Note that a change in slope is observed for n > 15 in the case of **poly-ZnT_2_isoP2** (Figure 2B). It might be related to the incorporation of supporting electrolyte, monomers as well as solvent molecules.

**Figure 3:**
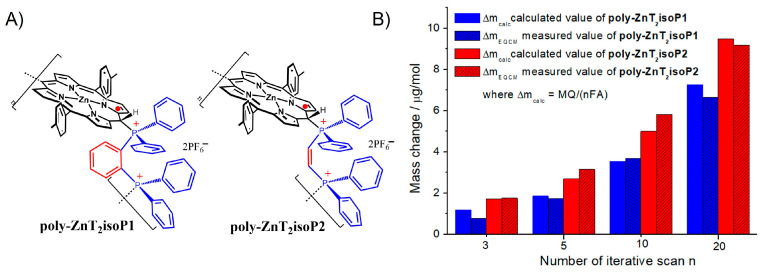
(**A**) Repeat unit are (isoZnT_2_P-^+^PPh_2_-HC=CH-PPh_2_^+^·2PF_6_^−^) and (isoZnT_2_P-^+^PPh_2_-Ph-PPh_2_^+^·2PF_6_^−^) for **poly-ZnT_2_isoP1** and **poly-ZnT_2_isoP2**, respectively. (**B**) The mass change calculated from Faraday’s law (Δm_calc_ = MQ/(nFA), where M is the molecular mass of the repeat unit, Q is the anodic charge measured from CV, n the number of electrons exchanged, 3 electrons and is the surface of the electrode) and compared with Δm_EQCM_ measured from EQCM data *vs.* the iterative scan numbers *n* (*n* = 3, 5, 10 and 20).

After 25 iterative scans, 7.81 µg.cm^−2^ of **poly-ZnT_2_isoP1** and 11.46 µg.cm^−2^ of **poly-ZnT_2_isoP2** are deposited. The repeat units are (isoZnT_2_P-^+^PPh_2_-HC=CH-PPh_2_^+^·2PF_6_^−^) and (isoZnT_2_P-^+^PPh_2_-Ph-PPh_2_^+^·2PF_6_^−^) for **poly-ZnT_2_isoP1** and **poly-ZnT_2_isoP2**, respectively (Figure 3).

The calculated surface coverages Γ, after *n* = 25 iterative scans between −1.0 V and +1.0 V *vs.* SCE, are 8.36 × 10^−9^ mol·cm^−2^ and 11.78 × 10^−9^ mol·cm^−2^ for **poly-ZnT_2_isoP1** and **poly-ZnT_2_isoP2**, respectively. The result showed that the mass deposited of poly-isoporphyrin copolymer obtained is higher in the case of 1,2-bis(diphenylphosphino)benzene which means that this ligand shows better reactivity toward the nucleophilic attack than *cis*-1,2-bis(diphenylphosphino)ethene. It may be due to the higher flexibility of the disphosphane *cis*-1,2-bis(diphenylphosphino)ethene where Z/E isomerization under illumination is possible. Such isomerization is not possible in the case of the rigid 1,2-bis(diphenylphosphino)benzene (**2**).

The mass change Δm_calc_ can also be calculated from Faraday’s law by using the anodic charge Q as well as the molecular mass of the repeat unit of the copolymer. The calculated values Δm_calc_ are close to the experimental measurement Δm_EQCM_ obtained from EQCM study matching well to the formation of the 1D copolymer (Figure 3B and Table S1).

### 2.3. X-ray Photoelectron Spectroscopy (XPS)

**Figure 4:**
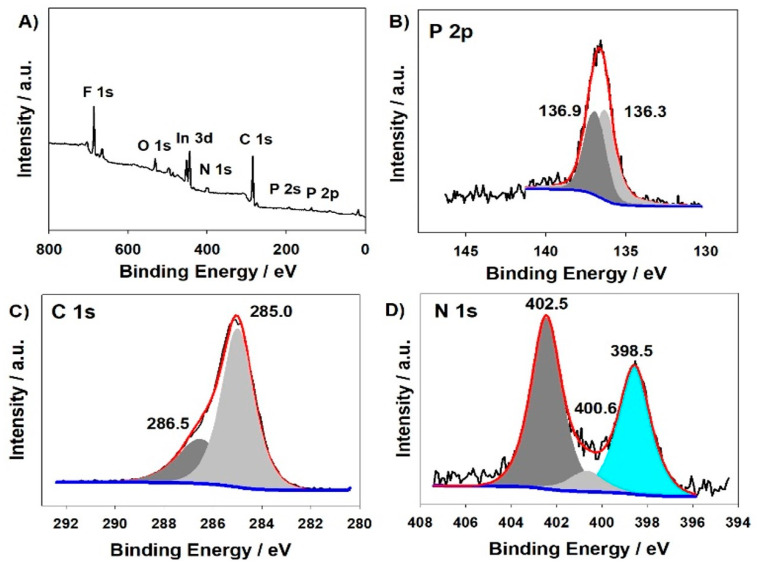
XPS spectra of the modified ITO electrodes with **poly-ZnT_2_isoP2** obtained after 25 iterative scans between −1.1 V and 1.0 V versus SCE. XPS spectra (**A**), P 2p (**B**), C 1s (**C**), N 1s (**D**).

The X-Ray photoelectron spectroscopy of **poly-ZnT_2_isoP2** was also investigated as shown in Figure 4. The analysis of the survey spectra confirms the presence of the isoporphyrin and diphosphonium subunits (Zn 2p3 at 1022.0 eV, N 1s, and C 1s peaks), while the signals for F 1s (686.7 eV) and for P 2p (136.9 eV and 136.3 eV) electrons stem from the incorporated counterion PF_6_^–^ as well as the phosphonium linkers (for the second P 2p signal at 136.3 eV). The C1s peaks are composed of two signals, 286.5 eV and 285.0 eV attributed to homo and hetero (connected to nitrogen) carbon atoms, respectively. The N1s peaks at 402.5 eV, 400.6 eV and 398.5 eV reveal the presence of three chemically different nitrogens due to the counter cation tetrabutylammonium, and the inner iminic nitrogen of the porphyrin. The whole XPS spectra are shown in Figure S3. Similar results have been obtained in the case of the second copolymer **poly-ZnT_2_isoP1** (Figure S2).

Analysis from the XPS data for the two copolymers can give the zinc-to-phosphorus atomic ratio, which is approximately 0.24 for the two copolymers. It is in agreement and very close to the theoretical ratio (0.25, one Zn per four P atoms, corresponding to the two phosphonium of the linker and the two PF_6_^−^ counter anions) in the case of the formation of the 1D copolymer where the repeat units are (iso**Zn**T_2_P-^+^**P**Ph_2_-HC=CH-**P**Ph_2_^+^·2**P**F_6_^−^) and (iso**Zn**T_2_P-^+^**P**Ph_2_-Ph-PPh_2_^+^·2**P**F_6_^−^) for **poly-ZnT_2_isoP1** and **poly-ZnT_2_isoP2**, respectively. Thus, it confirms the 1D copolymer formation as suggested by the EQCM measurements.

### 2.4. Electron Paramagnetic Resonance (EPR)

**Figure 5:**
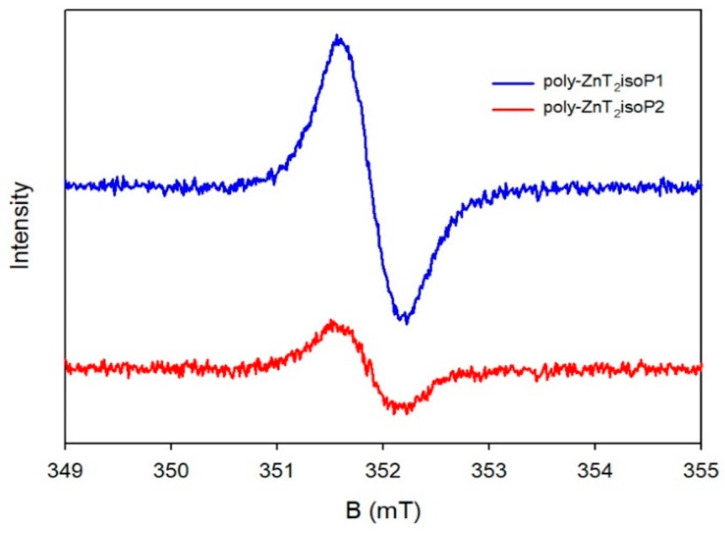
X-Band EPR spectrum in DMF of **poly-ZnT_2_isoP1** and **poly-ZnT_2_isoP2** at room temperature. The solution of copolymer was prepared by washing with 160 mL of DMF the covered ITO obtained using 25 scans between 1.1 V and +1.0 V versus SCE and -, v = 100 mV·s^−1^. In order to have enough solution, the operation was repeated three times.

The conspicuous central signal within these spectra confirms the presence of isoporphyrin-based free radicals (Figure 5), exhibiting a *g*-factor of 2.003 and a linewidth of approximately 0.6 mT [18]. Notably, an increase in the deposition concentration of the copolymers on the ITO led to a commensurate rise in the EPR signal strength. Typically, in the literature, isoporphyrins and their radical cationic forms are known to be highly reactive, prone to decomposition [19,20].

However, in this study, the isoporphyrin free radical films of **poly-ZnT_2_isoP1** and **poly-ZnT_2_isoP2** exhibited surprising stability in a solid state, even when exposed to the atmosphere, enduring up to 4 years. This remarkable stability can likely be attributed to the following factors: the electron-withdrawing groups playing a pivotal role in facilitating the reduction and stabilization of the radical cations, and the free radicals benefiting from the electronic delocalization effects of the porphyrin, in addition to the π–π interactions between molecules and the π–π stacking effects between the macrocycles.

### 2.5. UV–Visible–NIR Spectroscopy

**Figure 6:**
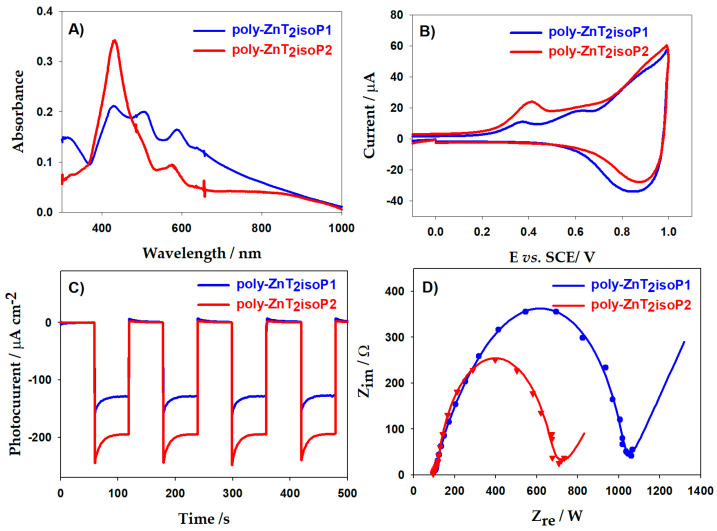
(**A**) UV–vis–NIR spectra of **poly-ZnT_2_isoP1** and of **poly-ZnT_2_isoP2**. (**B**) Cyclic voltammograms recorded for **poly-ZnT_2_isoP1** and **poly-ZnT_2_isoP2**. (**C**) Photoelectrochemical response. (**D**) ESI Nyquist of **poly-ZnT_2_isoP1** after 20 iterative scans and of **poly-ZnT_2_isoP2** for the film obtained after 10 iterative scans.

Figure 6A showed UV–visible–NIR spectra on ITO electrodes coated with the poly-isoporphyrin measured at various thicknesses. The absorption intensity increases with iterative scan number which showed same trend with EQCM results (Figure S4).

A typical UV–visible–NIR spectrum of **poly-ZnT_2_isoP1** obtained after electropolymerization between −1.1 and +1.0 V *vs.* SCE exhibited a split Soret absorption band at λ = 432 nm and λ = 505 nm. The bands were red shifted by 18 and 90 nm, respectively, compared to the **ZnT_2_P** monomer (Table 2).

The visible bands (Q bands), observed at 587 and 633 nm, are also red-shifted by 41 and 49 nm compared to ZnT_2_P, while for this poly-isoporphyrin, additional band in the NIR region were detected at 810 nm (Figure 6A). For **poly-ZnT_2_isoP2**, Soret absorption bands are at λ = 432 nm and λ = 497 nm, and Q bands are at 574 nm, 632 nm, also one additional absorption band in NIR region at 805 nm. This bathochromic shift may result from the electron withdrawing effect of the positive charge on the phosphonium cations.

The red shift observed in the Soret (B) and Q bands should be attributed to the nonplanar conformation of the macrocycle, as well as the electron-withdrawing effects of the pyridinium groups. Indeed, the red shift observed in the Soret (B band) and Q bands caused by the non-planarity of porphyrins are well documented in the literature [21,22,23,24]. Optical red shifts may be also attributed to the electron-withdrawing effects of the pyridinium groups. This phenomenon is explained by a greater destabilization of the highest occupied molecular orbitals (HOMOs) compared to the lowest unoccupied molecular orbitals (LUMOs), leading to narrower HOMO–LUMO gaps [25,26,27,28]. These spectral changes can also be interpreted by considering the intra- and intermolecular exciton coupling between the porphyrin macrocycles within the copolymer [29,30].

The splitting of the Soret band as well as the additional NIR band could be the result to the formation of the related radical isoporphyrin [16]. The weak absorption band in NIR region more pronounced for **poly-ZnT_2_isoP2** (Figure 6A and Figure S4) may because of the high delocalization of π-cation radicals of poly-isoporphyrin as well as the presence of the isoporphyrin subunits [16]. Similar bands have been already observed in the case of stable isoporphyrin monomer [31].

In Figure S4C, we observe results which seem contradictory to those obtained by the EQCM measurements if we only compare the maximum absorption intensity at λ = 432 nm. However, it can be noted that for **poly-ZnT_2_isoP1**, the splitting of the Soret absorption bands is more marked with a more marked widening of the bands. This is undoubtedly due to greater flexibility for **poly-ZnT_2_isoP1** where the spacer is the *cis*-1,2-bis(diphenylphosphonium)ethene. It is therefore difficult in this case to compare only the intensity of the Soret band at λ = 432 nm.

Interestingly, the broadening and the splitting of the Soret band as well as the presence of one additional band in the NIR between 750 nm and 1000 nm are expected to be advantageous to photovoltaic applications by extending the domain of solar light absorption.

### 2.6. Film Morphology (Atomic Force Microscopy, AFM)

**Figure 7:**
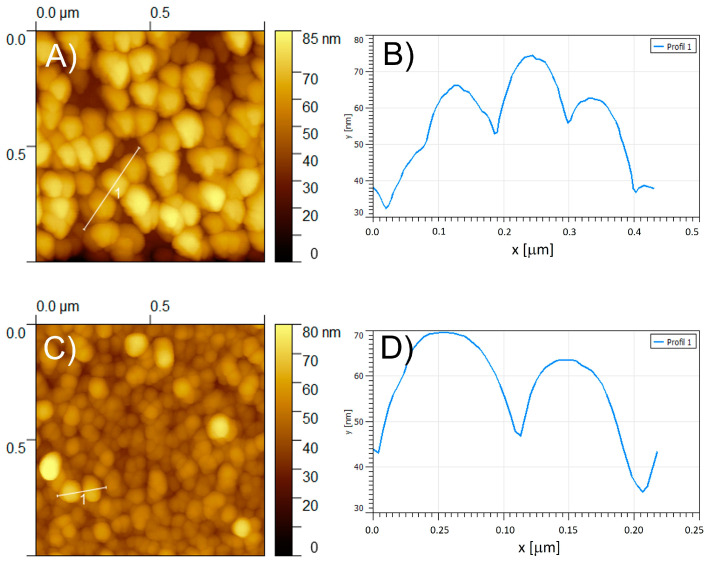
Tapping mode AFM topography of (**A**,**C**) of **poly-ZnT_2_isoP1** and **poly-ZnT_2_isoP2**, respectively. (**B**,**D**) Section analysis of the coils marked by a white line of **poly-ZnT_2_isoP1** and **poly-ZnT_2_isoP2**, respectively.

The obtained films were studied by scanning atomic force microscopy (AFM) (Figure 7A,C).

In a typical image of **poly-ZnT_2_isoP_1_**, the copolymer is seen on the surface as densely packed coils with an average diameter of approximately 80–150 nm and a height approximately 22.0 nm for the film produced after 10 iterative scans between −1.1 V and +1.0 V (Figure 6A). The rms surface roughness of the two films was estimated to be 14.2 nm for **poly-ZnT_2_isoP_1_**, based on calculations from an area of 1.0 μm^2^ with 10 scans (Figure 6).

The **poly-ZnT_2_isoP1** copolymer films obtained after higher iterative scan number exhibited comparable morphology but showed in several positions some aggregation of the coils accompanied by a larger value of the rms surface roughness (24.9 nm for *n* = 15). The formation of coil aggregates might be related to the change in slope of the deposited mass, observed from EQCM measurements in Figure 2B.

The **poly-ZnT_2_isoP2** films were also studied by AFM (Figure 7C,D). The **poly-ZnT_2_isoP2** copolymer appears on the surface yet again as tightly packed coils with an average diameter of ca. 80–100 nm, the height being approximately 24.0–33.0 nm for the film. The rms surface roughness of the films has been estimated at 7.6 nm for *n* = 10 scans (calculated from an area of 1.0 μm^2^).

### 2.7. Electrochemistry of the Copolymer

The cyclic voltammetry of electroactive polymers deposited on ITO surfaces have been characterized by electrochemical methods (Table 2). The CV curves shown in Figures S5 and S6 have been recorded for **poly-ZnT_2_isoP1** and **poly-ZnT_2_isoP2** with copolymer grown on the electrode surface for various potential scans (*n* = 1, 2, 3, 5, 10, 15, 20, 25 cycles) in a clean electrolytic solution containing only the solvent and the supporting electrolyte.

When porphyrin was substituted by such electron-withdrawing groups, the oxidation of the substituted isoporphyrins was more difficult than the oxidation of the corresponding unsubstituted porphyrins **ZnT_2_P** (Table 2).

In the anodic part, three successive waves are observed, the first wave is reversible while the two last waves are irreversible (Table 2). The first reversible processes were measured at +0.86 V and +0.88 V for **poly-ZnT_2_isoP1** and **poly-ZnT_2_isoP2**, respectively. This corresponded to the wave which increases in intensity during electropolymerization (Figure 1B,C). The reversible wave corresponds to the reversible oxidation of the isoporphyrin radical isoP^●^ giving the oxidized isoporphyrin isoP^+^.

The two irreversible oxidation peaks were observed after the reversible wave for **poly-ZnT_2_isoP1** and **poly-ZnT_2_isoP2** at 1.15 V and 1.39 V *vs.* SCE and at 1.17 V and 1.40 V *vs.* SCE, respectively (Figure S5).

In the cathodic domain, not well-resolved wave observed approximately –0.91 V *vs.* SCE and –0.87 V *vs.* SCE (peaks I or I’) for **poly-ZnT_2_isoP1** and **poly-ZnT_2_isoP2**, respectively, are attributed to the reduction of the phosphonium forming the corresponding radical (-^●^P(Ph)_2_-).

Furthermore, after the reduction of the phosphonium units, the additional wave measured at +0.42 V and +0.38 V for **poly-ZnT_2_isoP1** and **poly-ZnT_2_isoP2**, respectively (peak a or a’), which were also observed during electropolymerization (peaks a or a’) might correspond to the reaction between the radical phosphine –(Ph)_2_P^●^– formed after reduction of the phosphonium unit and the radical present on to the isoporphyrin isoP^●^.

Thus, the reduction of the diphosphonium generates one biradical which can react rapidly via radical coupling with the radical present onto the isoporphyrin (formation of the internal C-C bound between (-^●^P(Ph)_2_- and the IsoP^●^) as proposed in Scheme S3.

The peaks a or a’ might be due to the oxidation of the resulting compound obtained to regenerate the starting isoporphyrin radical-phosphonium material.

**Poly-ZnT_2_isoP1** and **poly-ZnT_2_isoP2** are highly stable even after 4 years, but at a higher applied potential than the first oxidation wave, which is reversible, at 0.86 V and 0.88 V, respectively, for instance at an applied potential of 1.60 V, fully decomposition of the film occurs leading to the fully removal of the film from the ITO slides (Figure S5, right).

### 2.8. Electrochemical Impedance Spectroscopy (EIS) and Photoelectrochemical Properties

The electrochemical and photoelectrochemical properties of this two poly-isoporphyrins are investigated and compared (Figure 6B,C, and Figures S7 and S8).

The typical current–potential curves were obtained by photocurrent measurements (Figures S7 and S8). For the best photocurrent value of both **poly-ZnT_2_isoP1** (20 scans) and **poly-ZnT_2_isoP2** (10 scans) samples, the amount of deposited copolymer on ITO substrate is approximately the same (ca. 6.5 µg/cm^2^), thus enabling the comparison of their (photo)electrochemical properties.

Compared with Figure S7, the photocurrent response depends on the number of deposition scans. The magnitude of the photocurrent increased from *n* = 1 to *n* = 10, and then as decreased with higher deposition scans of **poly-ZnT_2_isoP1**. The same trend is observed for **poly-ZnT_2_isoP2**.

The electrical properties of copolymer films were studied by electrochemical impedance spectroscopy (EIS), which is widely used in the investigation of dye sensitized solar cell. Under light illumination, the **poly-ZnT_2_isoP2** showed a better conductivity than **poly-ZnT_2_isoP1** according to the smaller semi-circle (Figure 6D and Figure S9). This appearance seems to be correlated with the magnitude of the photocurrent measured under visible illumination. All the results showed that the **poly-ZnT_2_isoP2** had a better photocurrent generation under visible illumination (Figure 6C and Figure S9).

The Nyquist plots of a typical impedance spectra measured for **poly-ZnT_2_isoP1** and **poly-ZnT_2_isoP2** at 0 V in 5 mM I_3_^−^/0.5 M I^−^ aqueous solution with different scan numbers (*n* = 1, 2, 3, 5, 10, 15, 20 and 25) are plotted in Figure S9. The Nyquist diagrams can be fitted with an equivalent circuit composed of two parallel RC circuits in series. One RC circuit can be attributed to the charge transfer resistance R_ct_ of the I^−^/I_3_^−^ species at the ITO/solution interface in parallel with the interfacial capacitance C_i_, while the other RC circuit also is due to the ion transport resistance R_f_ within the copolymer film in parallel with the C_f_ capacitance of the film. The evolution of the R_ct_ and R_f_ values with the number of polymerization scan number under and without illumination shown in Figure S10.

Under illumination, the charge transfer resistance decreased compared to ones measured in the dark conditions, illustrating the shift of the open circuit potential and then the photochemical activity of copolymers when illuminated. The charge transfer resistance (R_ct_) decreases with scan number indicating that the presence of the copolymer film is catalyzing the electron transfer kinetics between I^−^/I_3_^−^ and the ITO electrode. Meanwhile, the R_f_ tends to increase with the number of electrodeposition scans n due to the increase in film thickness due to the ionic resistance into the copolymer film. Thus, the lowest total charge transfer resistance is obtained with 20 scans for **poly-ZnT_2_isoP1** and 15 scans for **poly-ZnT_2_isoP2**, which showed almost the same trend with photocurrent. It suggests that the photocurrent efficiency is dependent on charge transfer resistance. For **poly-ZnT_2_isoP1** at 20 scans under light illumination, R_ct_ is 397 ohms versus R_f_ of 534 ohms. For **poly-ZnT_2_isoP2**, at 15 scans under light illumination, R_ct_ is 456 ohms versus R_f_ of 18 ohms. Thus, poly- **poly-ZnT_2_isoP2** possessed better conductivity with similar deposited mass than **poly-ZnT_2_isoP1**.

**Figure 8:**
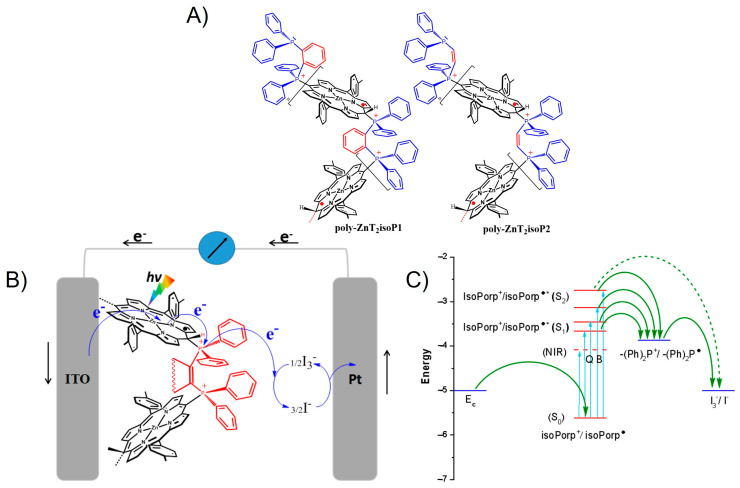
(**A**) The structures of **poly-ZnT_2_isoP1** and **poly-ZnT_2_isoP2**. (**B**,**C**) Schematic illustrations of the energy level diagram for **poly-ZnT_2_isoP1** showing electron transfer processes in H_2_O containing I_3_^−^ 5 mmol·L^−1^ and I^−^ 0.5 mol·L^−1^ (-(Ph)_2_P^+^- = di(phenylphosphonium); Porp = porphyrin).

To investigate the electron transfer mechanism in the system, the energies of the relevant electronic states have been estimated. In the case of copolymer **poly-ZnT_2_isoP1**, an energy level diagram can be built using the oxidation potential of isoporphyrin subunit and the reduction potential of phosphonium subunits, together with the optical absorption spectra of copolymer films (Figure 8). It describes the thermodynamics for spectral sensitization of the ITO electrode. The LUMO levels of the excited **poly-ZnT_2_isoP1** and **poly-ZnT_2_isoP2** species can be roughly estimated by subtracting the excitation energy of the Soret or Q bands from the HOMO level energies. The levels of the excited isoporphyrin, isoPorph^+^/isoPorph^●^*, are calculated from the absorption spectra of the film and the wavelength of the Soret (B), Q and NIR bands. The ground state isoporphyrin (isoPorph^+^/isoPorph^●^) is calculated from the oxidation potential of the isoporphyrin giving for instance in the absolute scale –5.60 eV for the **poly-ZnT_2_isoP1** film. The energy level of I_3_^−^/I^–^ on the absolute scale is –5.00 eV. The energy Level of **poly-ZnT_2_isoP2** corresponding to the band in the NIR region has also been indicated in the energy diagram (Figure 8). Under illumination at 0 V, the photon absorption by the (iso)porphyrin entities generate an electron hole pair in the copolymer. The electron is transferred from the excited (iso)porphyrins to the phosphonium subunit acting as a relay for the electron transfer forming the radical phosphane. This radical phosphane gives in turn the electron to the I_3_^−^, which is reduced into I^−^ which, is reoxidized at the counter-electrode. The oxidized (iso)porphyrins are regenerated by an electron transfer from the ITO substrate. The I_3_^−^ reduction is in competition with the recombination of the photogenerated electron-hole pair. Thus, fast electron transfer kinetics between the ITO and the oxidized (iso)porphyrins and/or between the excited (iso)porphyrins and the I_3_^−^ are crucial for the efficient photocurrent generation.

## 3. Materials and Methods

### 3.1. Reagents

All solvents were of reagent-grade quality and used without further purification. Most common laboratory chemicals were reagent grade, purchased from commercial sources and used without further purification. The zinc-5,15-ditolylporphyrin (ZnT_2_P) was purchased from SAS PorphyChem^®^. *cis*-1,2-bis(diphenylphosphino)ethene (**1**) or 1,2-bis(diphenylphosphino)benzene (**2**).

### 3.2. Electrochemistry

Voltametric data were collected using a standard three-electrode setup with a PARSTAT 2273 potentiostat. The electrolyte solution was a mixture of CH_3_CN and 1,2-C_2_H_4_Cl_2_ (3:7) containing 0.1 mol·L^−1^ of tetrabutylammonium hexafluorophosphate (NBu4PF_6_). Single-side-coated indium-tin-oxide (ITO, SOLEMS, 25–35 Ω/cm^2^) electrodes with a surface area of 1 cm^2^ served as the working electrode, while a platinum wire functioned as the auxiliary electrode. The reference electrode was a saturated calomel electrode, connected to the solution via a junction bridge filled with the electrolyte. Electrochemical impedance spectroscopy (EIS) measurements were conducted with a sinusoidal voltage excitation of 10 mV amplitude across a frequency range of 100 kHz to 100 mHz. Additionally, ITO electrodes were employed to obtain UV–vis spectra of the electrochemically deposited copolymers using an Agilent 8453 spectrophotometer.

### 3.3. Electropolymerization

Electropolymerization was performed under an argon atmosphere using a solution of 0.1 mol·L^−1^ TBAPF_6_ in 1,2-C_2_H_4_Cl_2_/CH_3_CN (7/3) containing 0.25 mmol·L^−1^ of **ZnT_2_P** and 0.25 mmol·L^−1^ of either *cis*-1,2-bis(diphenylphosphino)ethene (**1**) or 1,2-bis(diphenylphosphino)benzene (**2**), within a voltage range of −1.1 to 1.0 V *vs.* SCE.

The formation of the copolymer can be carried out either by applying a constant potential (chronoamperometry), or by iterative scanning between two potentials (cyclic voltammetry). Chronoamperometry works but gives less controlled deposits and less homogeneous materials in terms of film thickness. It must be noted that electropolymerization at controlled potential is only possible beyond the first oxidation potential of the porphyrin **ZnT_2_P**, i.e., 0.79 V *vs.* SCE and below the oxidation potentials of the diphosphine ligand, namely the *cis*-1,2-bis(diphenylphosphino)ethene (**1**) or 1,2-bis(diphenylphosphino)benzene (**2**), at 1.37 V and 1.68 V *vs.* SCE, respectively. Furthermore, by applying a constant potential between 0.79 V and 1.37 V or 1.69 V *vs.* SCE for *cis*-1,2-bis(diphenylphosphino)ethene (**1**) or 1,2-bis(diphenylphosphino)benzene (**2**), respectively, an accumulation of the released H^+^ occurs which can induce a demetalation of the metalloporphyrin **ZnT_2_P**, follow by a protonation of the free base obtained H_2_T_2_P, leading to the formation of protonated porphyrin which will stop the reaction, these protonated porphyrins being much more difficult to be oxidized.

The cyclic voltammetry method over a voltage range of −1.1 to 1.0 V *vs.* SCE gave better results from the deposit quality point of view with a more homogeneous deposit thickness across the entire sample. The potential of 1.0 V was chosen to be sufficiently higher than the first oxidation potential of **ZnT_2_P** (0.79 V) and sufficiently lower than the oxidation potentials of *cis*-1,2-bis(diphenylphosphino)ethene (**1**) and 1,2-bis(diphenylphosphino)benzene (**2**). The scan rate v = 100 mV·s^−1^ allowed to obtain good-quality films with a preparation time for each sample that is not too long. The advantage of iterative scanning between −1.1 V and 1.0 V *vs.* SCE in cyclic voltammetry mode also makes it possible to consume and reduce the H^+^ released during the substitution by forming H_2_(g).

Below −1.1 V, a reduction in porphyrin macrocycle also occurs, inducing the formation of poor-quality film. Control of the optical spectra of the solution shows that no demetallation of the **ZnT_2_P** porphyrin is noticed in the case of iterative scan between −1.1 V to 1.0 V *vs.* SCE.

ITO electrodes with a surface area of 1 cm^2^ were used as the working electrode. Each copolymer underwent 20 iterative scans. Following electropolymerization, the modified working electrodes were rinsed with CH_3_CN and then with CH_2_Cl_2_ to remove any residual monomers and conducting salt from the deposited films.

### 3.4. Electrochemical Quartz Cristal Microbalance (EQCM)

A QCA-922 system (SEIKO EG&G instrument) combined with a Versa STAT 3 was employed for simultaneous electrochemical quartz crystal microbalance (EQCM) and cyclic voltammetry measurements. The electrochemical cell was assembled inside a glove box, using an ITO AT-cut quartz crystal resonator (mirror-finished, resonant frequency: 9.08 MHz ± 50 kHz, A = 0.2 cm^2^, SEIKO EG&G., Ltd.) as the working electrode, a platinum wire as the counter electrode, and an Ag/AgCl wire as a quasi-reference electrode. The solution used for electropolymerization was identical to that used for the copolymers with the larger ITO electrode. Iterative scans were performed at a scan rate of 100 mV·s^−1^ at room temperature, with simultaneous recording of the quartz resonance frequency. The change in quartz resonance frequency (Δf) was converted into mass change (Δm) using Sauerbrey’s equation (Equation (1)):

∆f = −2f_0_^2^∆m/A(µ·ρ)^1/2^ (1)

where μ is the shear modulus of quartz (2.947 × 10^11^ g·cm^−1^·s^−2^), A is the working area (0.2 cm^2^) of the ITO quartz crystal resonator, and f_0_ is the resonant frequency of the fundamental mode, and ρ is the density of the crystal (2.684 g/cm^3^).

### 3.5. X-ray Photoelectron Spectroscopy

XPS experiments were conducted using a RBD upgraded PHI-5000C ESCA system (Perkin-Elmer) with Mg Kα radiation (hν = 1253.6 eV) or Al Kα radiation (hν = 1486.6 eV). Typically, the X-ray anode operated at 250 W, with a high voltage of 14.0 kV and a detection angle of 54°. The pass energy was set at 23.5, 46.95, or 93.90 eV to ensure adequate sensitivity and resolution. The base pressure in the analyzer chamber was approximately 5 × 10^−8^ Pa. Samples were pressed into self-supported disks (10 × 10 mm), mounted on a sample holder, and then transferred into the analyzer chamber. Both the full spectra (0–1100 eV) and the high-resolution narrow spectra of all elements were recorded using an RBD 147 interface (RBD Enterprises, Briarwood, NY, USA) with Auger Scan 3.21 software. Binding energies were calibrated using contaminant carbon (C1s = 284.6 eV). Data analysis was performed using either RBD Auger Scan 3.21 software from RBD Enterprises or XPS Peak 4.1 provided by Raymund W.M. Kwok (The Chinese University of Hong Kong, China).

### 3.6. Atomic Force Microscopy (AFM)

Atomic force micrographs (AFM) measurements have been conducted directly on the ITO surfaces using a NX10 Park Instrument in the tapping mode under ambient conditions. Silicon cantilevers Bruker with a spring constant of 40 N/m and a resonance frequency in the range of 300 kHz have been used. The scanning rate was 1.0 Hz.

### 3.7. EPR Measurements

CW EPR experiments were performed using an X-band (9–10 GHz) Bruker ESP300 spectrometer equipped with a high-sensitivity resonator (ER4119HS, Bruker) at room temperature.

### 3.8. Photoelectrochemistry

Photoelectrochemical measurements were conducted in a three-electrode electrochemical cell, with glass-ITO electrodes covered by the copolymer thin film as the photocathode, a Pt plate as the counter-electrode, and a Pt wire as the pseudo-reference electrode. The photoelectrochemical responses of the films were obtained through on–off light illumination in a 0.5 M I^−^/5 mM I_3_^−^ aqueous solution using a 300 W Xe arc lamp. The I^−^ and I_3_^−^ anions worked as redox mediators. A water filter was used to block IR radiation, and long pass filters at either λ > 405 nm or λ > 800 nm were employed to isolate visible-NIR or NIR radiation, respectively. Current–potential curves were recorded both in the dark and under illumination using a PARSTAT 2273 potentiostat with linear sweep voltammetry at a scan rate of 10 mV·s^−1^. EIS spectra were acquired under illumination by applying a sinusoidal potential modulation (10 mV over the Open Circuit Photopotential) across frequencies ranging from 0.1 to 100,000 Hz.

In photoelectrochemistry and voltametric measurements have been performed with a standard three-electrode system using a PARSTAT 2273 potentiostat. Glass carbon or single-side-coated indium-tin-oxide (ITO, SOLEMS, 25–35 Ω·cm^−2^) electrodes, with a surface of about 1 cm^2^ has been used as working electrode. A platinum wire has been used as an auxiliary electrode. The reference electrode was a saturated calomel electrode. It was electrically connected to the solution by a junction bridge filled with the electrolyte. Photoelectrochemical responses for films were obtained by on–off light illumination of a 300 W Xe Arc lamp (with λ > 405 nm long pass filter). Solutions were prepared by bubbling with argon (Ar–U, from Air Liquide) in order to have deaerated prior to illumination. All experiments were conducted at room temperature using a water filter to block IR radiation, ensuring that the solution temperature did not increase by more than 1 degree during light exposure. UV–vis absorption spectra were recorded using a Hewlett-Packard HP 8453 diode array spectrophotometer, which operated with a resolution of 2 nm.

## 4. Conclusions

In summary, copolymer films have been prepared by the electro-oxidation of 5,15-ditolylporphyrin (ZnT_2_P) in the presence of various types of di(phenylphosphane) ligands. Electrogenerated radical cation porphyrin is a powerful electrophile which can rapidly react to form copolymer containing stable isoporphyrin subunits (**poly-ZnT_2_isoP**^●^). The achieved copolymers were characterized by UV–vis–NIR spectroscopy, and X-ray photoelectron spectroscopy. Mechanisms of electrochemical routes to these electroactive copolymers have been discussed as well as the description of the unusual redox properties of copolymers containing stable isoporphyrin.

The photocurrent measurements under visible–NIR light irradiation show that **poly-ZnT_2_isoP2** thin films exhibit significantly better performance than **poly-ZnT_2_isoP1**. However, the photovoltaic performances reach an optimum depending on the number of electropolymerization potential scan number. The best performances for **poly-ZnT_2_isoP1** and **poly-ZnT_2_isoP2** are obtained for *n* = 20 and 10, respectively. Therefore, the control of the thickness of the copolymer films is of great importance to optimize the generation of photocurrent under visible illumination. The copolymer in the solid state could be stored for over several months and even one year without any degradation under ambient conditions in air.

## Figures and Tables

**Table 1:** UV–visible spectral data for ZnT_2_P, *cis*-1,2-bis(diphenylphosphino)ethene and 1,2-bis(diphenylphosphino)benzene in 1,2-CH_2_Cl_2_, **poly-ZnT_2_isoP1**, **poly-ZnT_2_isoP2** on optical transparent ITO electrodes. Under bracket: molar extinction coefficient (ε/10^3^ L·M·cm^−1^).

Compound	Soret Band/nm	Q and NIR Bands/nm	π–π* Band/nm
**ZnT_2_P** ^1^	415 (405.4)	546 (18.9), 584 (4.9)	
*cis*-1,2-bis(diphenyl-phosphino)ethene ^1^			214 (44.9), 257 (19.7)
1,2-bis(diphenyl-phosphino)benzene ^2^			214 (58.7), 278 (17.9)
**poly-ZnT_2_isoP1** ^3^	432, 505	587, 633, 810	
**poly-ZnT_2_isoP2** ^4^	432, 497	574, 632, 805	

^1^ Due to the steric hindrance of *cis*-1,2-bis(diphenylphosphino)ethene (**1**) or 1,2-bis(diphenylphosphino)benzene (2), coordination between the diphosphine ligand and the Zn is not observed giving a similar optical spectrum with and without diphosphine **1** or **2** with **ZnT_2_P**; ^2^ in CH_2_Cl_2_; ^3^ Copolymers obtained by iterative scan between −1.1 V and +1.0 V *vs.* SCE onto ITO; ^4^ Copolymers obtained by iterative scan between −1.1 V and +1.0 V *vs.* SCE onto ITO.

**Table 2:** Electrochemical data for ZnT_2_P, *cis*-1,2-bis(diphenylphosphino)ethene) and 1,2-bis(diphenylphosphino)benzene, **poly-ZnT_2_isoP1** and **poly-ZnT_2_isoP2**.

Compounds	Ring Oxidation				Reduction of the -P^+^(Ph)_2_-	Ring Reduction		Oxidation of Phosphane -P^+●^(Ph)_2_−/ -P(Ph_2_)-
				peak a or a’	peak I or I’			
**ZnT_2_P ^1^**		1.08 (150)	0.79 (90)			−1.41 (160)	−1.84 (170)	
***cis*-1,2-bis(diphenyl** **phosphino)ethene)**								1.37 ^irr^
**1,2-bis(diphenyl** **phosphino)benzene**								1.68 ^irr^
**poly-ZnT_2_isoP1**	1.39 ^irr^	1.15 ^irr^	+0.86 (250)	+0.42 ^irr^	−0.91 ^irr^			
**poly-ZnT_2_isoP2** **^●^**	1.40 ^irr^	1.17 ^irr^	+0.88 (196)	+0.38 ^irr^	−0.87 ^irr^			

^1^ Potentials in V *vs.* SCE were obtained from cyclic voltammetry in 1,2-C_2_H_4_Cl_2_ with 0.1 mol·L^−1^ TBAPF_6._ Scan rate = 100 mV·s^−1^. Working electrode: ITO, S = 1 cm^2^. The given half-wave potentials are equal to E_1/2_ = (E_pa_+ E_pc_)/2. Under bracket: △E_p_ = |E_pa_-E_pc_|. Please note that the potential oxidation of the porphyrin did not change in the presence of *cis*-1,2-bis(diphenylphosphino)ethene (**1**) or 1,2-bis(diphenylphosphino)benzene (**2**). ^irr^ = irreversible.

## Data Availability

Data are contained within the article and the Supplementary Materials.

## Supplementary Materials

The following supporting information can be downloaded at: https://www.mdpi.com/article/10.3390/molecules29133056/s1, Scheme S1: The electropolymerization mechanism proposed to form the **poly-ZnT_2_isoP1** with the zinc 5,15-ditolylporphyrin (**ZnT_2_P**) and *cis*-1,2-bis(diphenylphosphino)ethene. Scheme S2: The electropolymerization mechanism proposed to form the poly-ZnT_2_isoP2 with the zinc 5,15-ditolylporphyrin (**ZnT_2_P**) and 1,2-bis(diphenylphosphino)benzene. Scheme S3: Proposed reactivity after reduction of the diphosphonium reduction (peak I or I’) in the case of **poly-ZnT_2_isoP1**. Figure S1: (A) Electrochemical quartz crystal microbalance measurements (Δ*m*) and consecutive cyclic voltammograms (first 25 scans) of **poly-ZnT_2_isoP1** with 1,2-bis(diphenylphosphino)ethene ligand. (B) Mass change (Δ*m*) of the first 25 scans calculated from Sauerbrey’s equation versus the number of scan n of **poly-ZnT_2_isoP1**. Figure S2: XPS spectra of the modified ITO electrodes with **poly-ZnT_2_isoP1** obtained with *cis*-1,2-bis(diphenylphosphino)benzene after 25 iterative scans between −1.1 V and 1.0 V versus SCE. XPS full spectra (A), C 1s (B), N 1s (C), Zn 2p (D), P 2p (E), F 1s (F) O 1s (G). Figure S3: XPS spectra of the modified ITO electrodes with poly-ZnT_2_isoP2 obtained with 11,2-bis(diphenylphosphino)ethene after 25 iterative scans between −1.1 V and 1.0 V versus SCE. XPS full spectra (A), C 1s (B), N 1s (C), Zn 2p (D), P 2p (E), F 1s (F) O 1s (G). Figure S4: UV–vis spectra of A) **poly-ZnT_2_isoP1** and B) **poly-ZnT_2_isoP2** obtained after n iterative scans (*n* = 1, 2, 3, 5, 10, 15, 20 and 25) on ITO. C) Plot of the intensity of the absorbance of the Soret band (432 nm) versus the iterative scan number between −1.1 V and +1.00 V versus SCE. Figure S5: Cyclic voltammograms of **poly-ZnT_2_isoP1** and **poly-ZnT_2_isoP2** obtained after *n* = 10 scans using iterative scan between −1.1 V and +1.0 V in CH_3_CN/1,2-C_2_H_4_Cl_2_ (3/7) with 0.1 M TBAPF_6_. WE: ITO. S = 1 cm^2^. *v* = 0.1 V·s^−1^. Right: Pictures of **poly-ZnT_2_isoP1** and **poly-ZnT_2_isoP2** copolymer films deposited at 25 scans between −1.1 and 1.0 V on the ITO and after oxidization to an applied potential of 1.6 V on the ITO. Figure S6: Cyclic voltammograms of (A) poly-ZnT_2_isoP1 between −1.0 V and +1.0 V and (B) poly-ZnT_2_isoP2 after *n* = 1, 3, 5, 10, 15 and 20 scans between −1.1 V and +0.9 V in CH_3_CN/1,2-C_2_H_4_Cl_2_ (3/7) with 0.1 M NBu_4_PF_6_. WE: ITO. S = 1 cm^2^, *v* = 0.1 V·s^−1^. Irreversible peaks not labelled in anodic part correspond to the oxidation of the π-ring of the macrocycle. Figure S7: Photoelectrochemical response of **poly-ZnT_2_isoP1** (black line) and of **poly-ZnT_2_isoP2** (red line) obtained with *n* = 1, 2, 3, 5, 10, 15, 20 and 25 iterative scans. Figure S8: (A) Current–potential curves of **poly-ZnT_2_isoP1** (obtained with *n* = 20 iterative scans between −1.0 V and +1.0 V *vs.* SCE) and **poly-ZnT_2_isoP2** (obtained with *n* = 10 iterative scans between −1.1 V and +1.0 V *vs.* SCE) thin films on ITO electrodes in 0.5 M I^−^/5 mM I_3_^−^ aqueous solution in the dark or under visible illumination. (B) and (C) Photoelectrochemical response of **poly-ZnT_2_isoP1** and **poly-ZnT_2_isoP2** films obtained with *n* = 1, 2, 3, 5, 10, 15, 20 or 25 iterative scans. Measurements have been obtained under on–off light illumination from a 300 W Xe arc lamp (with λ > 385 nm long pass filter) in I_3_^−^ 5 mmol·L^−1^ and I^−^ 0.5 mol·L^−1^ aqueous solution. BIAS potential: 0.00 V *vs.* OCP. Figure S9: (A) ESI Nyquist of **poly-ZnT_2_isoP1** and (B) **poly-ZnT_2_isoP2**. Measurements have been obtained in the dark and in H_2_O containing I_3_^−^ 5 mmol·L^−1^ and I^−^ 0.5 mol·L^−1^. BIAS potential: 0.00 V *vs.* OCP. Figure S10: Plot of the Log(R) versus n (number of iterative scan, v = 100 mVs^−1^) for (A) **poly-ZnT_2_isoP1** and (B) **poly-ZnT_2_isoP2**. Measurements have been obtained in H_2_O under on (red line) and off light (black line) illumination from a 300W Xe arc lamp (with λ > 385 nm long pass filter) in containing I_3_^−^ 5 mmol·L^−1^ and I^−^ 0.5 mol·L^−1^. BIAS potential: 0.00 V *vs.* OCP. (R_ct_ = charge transfer resistance at the ITO/copolymer interface (triangle plot with solid line), R_f_ = the charge transfer resistance of films (square plot with dotted line). Table S1: Mass change calculated from CV and Faraday’s law compared with the masse change measured from EQCM during CV measurements during electropolymerization for **poly-ZnT_2_isoP1** and **poly-ZnT_2_isoP2**.
