# Supplementary material for: Stable Radical Isoporphyrin Copolymer Prepared with Di(phenylphosphane)
Source: Molecules. 2024 Jun 27;29(13):3056. doi: 10.3390/molecules29133056 (PMC11243264; doi:10.3390/molecules29133056)
Supplement: Supplementary file 1 [file molecules-29-03056-s001.zip › molecules-3065259-supplementary.pdf]

Supplementary data

Stable radical isoporphyrin copolymer prepared with di(phenylphosphane)

Yiming Liang [1,2], Antoine Bonnefont [1,3], Vasilica Badets [2], Corinne Boudon [2], Michel Goldmann [4,5], Guillaume Diot [5], Sylvie Choua [2], Nolwenn Le Breton [2], Laurent Ruhlmann [2,*]

1 Zhengzhou Research Base, National Key Laboratory of Cotton Bio-Breeding and Integrated Utilization, School of Agricultural Sciences, Zhengzhou University, Zhengzhou 450001, China. liangyiming@zzu.edu.cn
2 Université de Strasbourg, Institut de Chimie, UMR CNRS 7177, 4 Rue Blaise Pascal, CS 90032, 67081, Strasbourg Cedex, France.
3 LEPMI, Université 589 Grenoble Alpes, Université Savoie Mont Blanc, CNRS, 590 Grenoble INP, F-38000 Grenoble, France.
4 Institut des Nanosciences de Paris, UMR CNRS 7588, Sorbonne Université, 4 Place Jussieu, Boîte Courrier 840, F - 75252, Paris, France.
5 Université Paris Cité, 45 Rue des Saints Pères, F - 75006, Paris, France.
* Correspondence: lruhlmann@unistra.fr

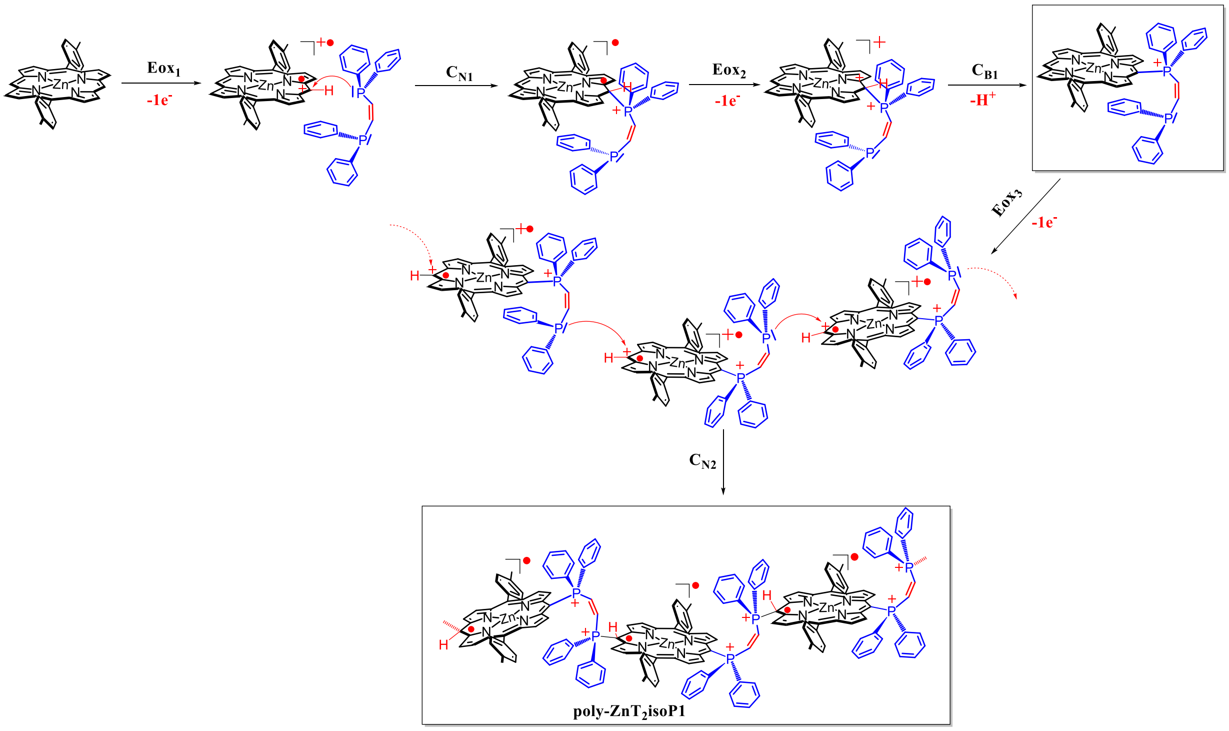

Scheme S1 The electropolymerization mechanism proposed to form the **poly-ZnT$_2$isoP1** with the zinc 5,15-ditolylporphyrin (ZnT$_2$P) and *cis*-1,2-bis(diphenylphosphino)ethene.

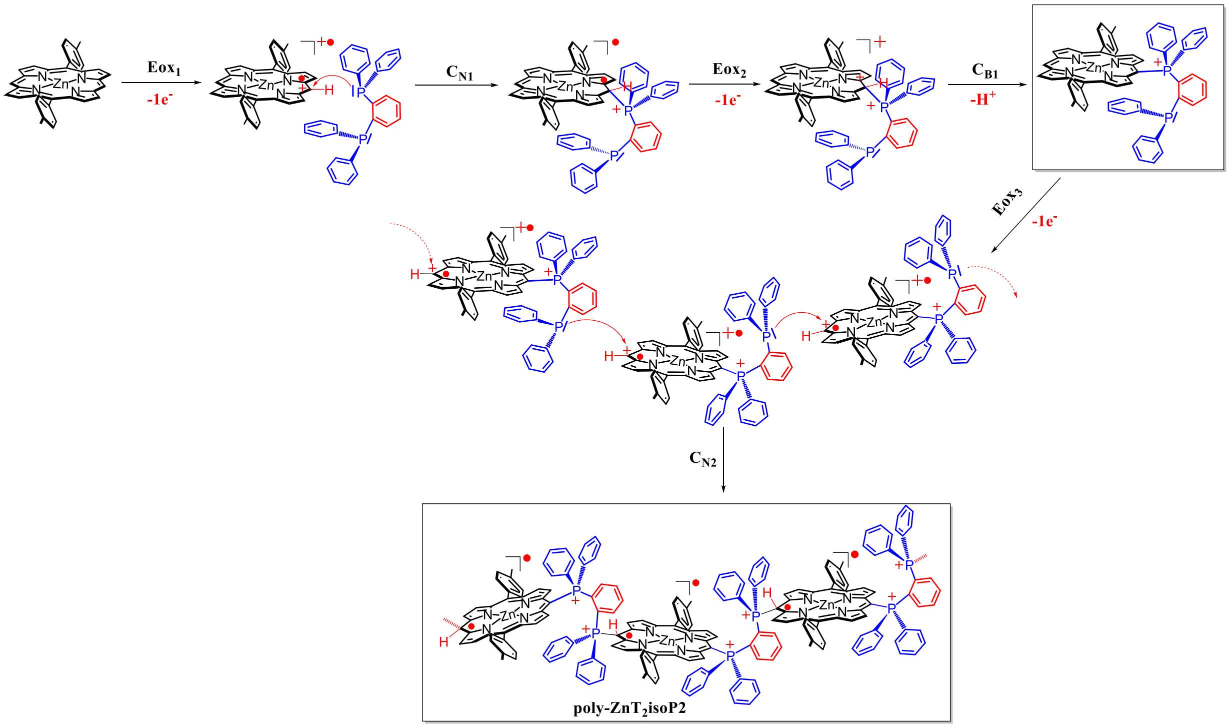

Scheme S2 The electropolymerization mechanism proposed to form the **poly-ZnT$_2$isoP2** with the zinc 5,15-ditolylporphyrin (ZnT$_2$P) and 1,2-bis(diphenylphosphino)benzene.

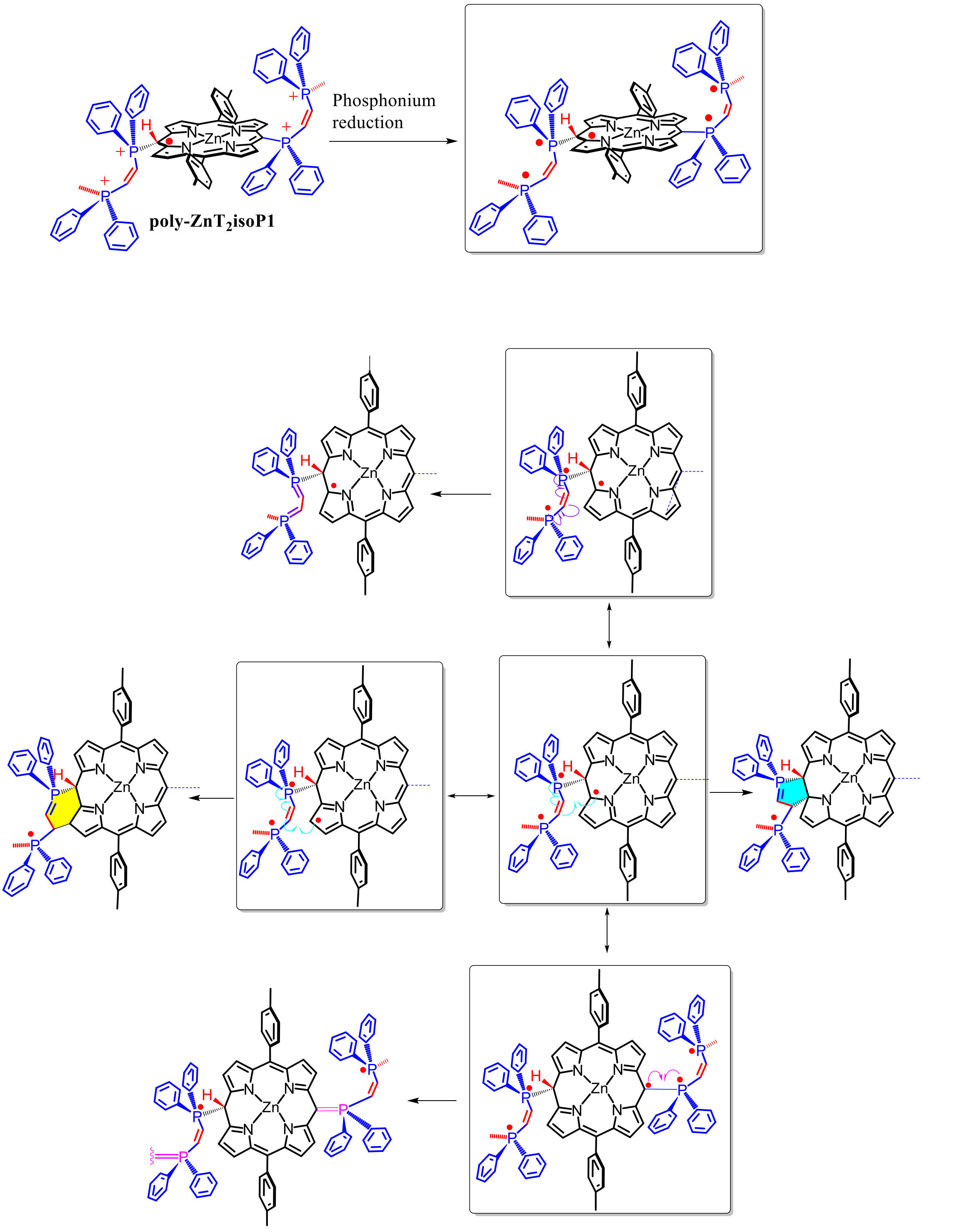

Scheme S3 Proposed reactivity after reduction of the diphosphonium reduction (peak I or I') in the case of **poly-ZnT$_2$isoP1**.

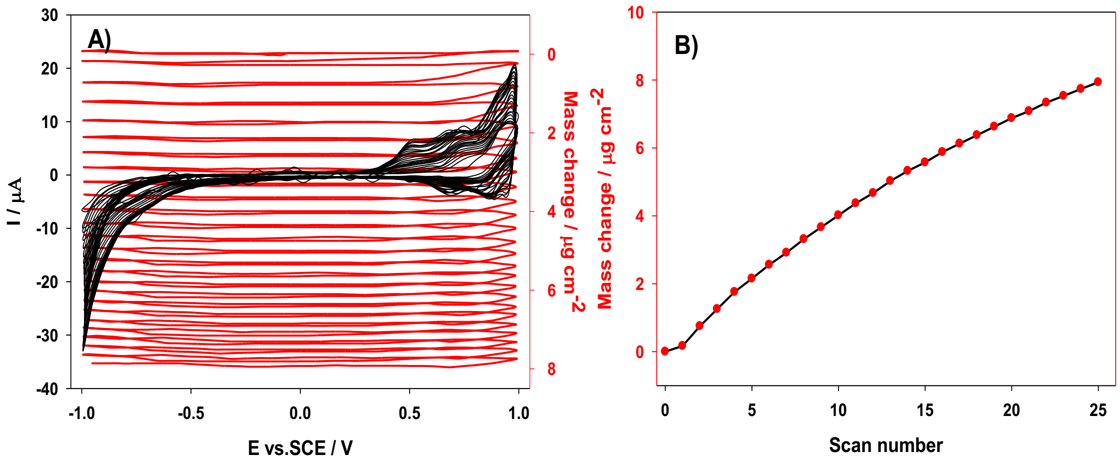

Fig. S1 A) Electrochemical quartz crystal microbalance measurements (*Δm*) and consecutive cyclic voltammograms (first 25 scans) of **poly-ZnT$_2$isoP1** with 1,2-bis(diphenylphosphino)ethene ligand. B) Mass change (*Δm*) of the first 25 scans calculated from Sauerbrey's equation *versus* the number of scan n of **poly-ZnT$_2$isoP1**.

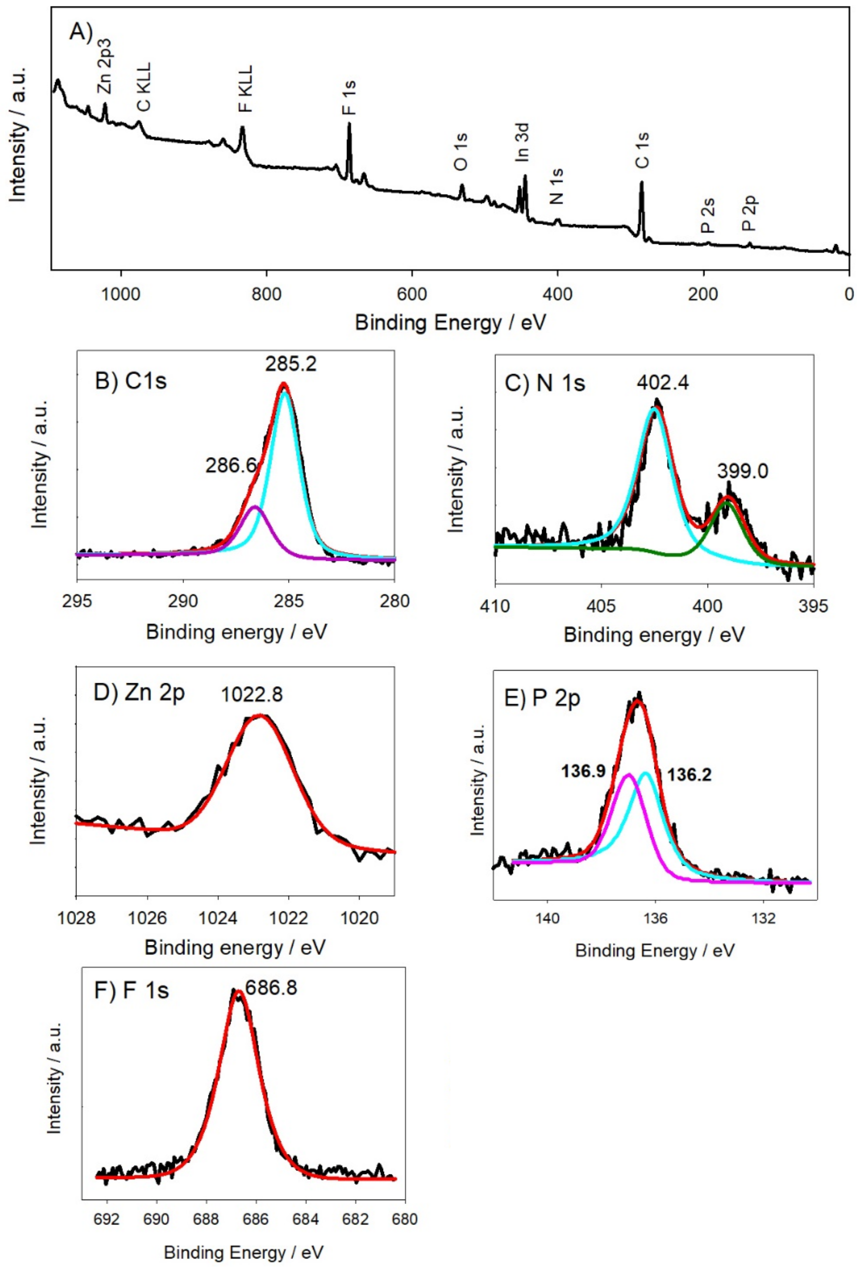

Fig. S2 XPS spectra of the modified ITO electrodes with **poly-ZnT$_2$isoP1** obtained with cis-1,2-bis(diphenylphosphino)benzene after 25 iterative scans between -1.1 V and 1.0 V *versus* SCE. XPS full spectra (A), C 1s (B), N 1s (C), Zn 2p (D), P 2p (E), F 1s (F).

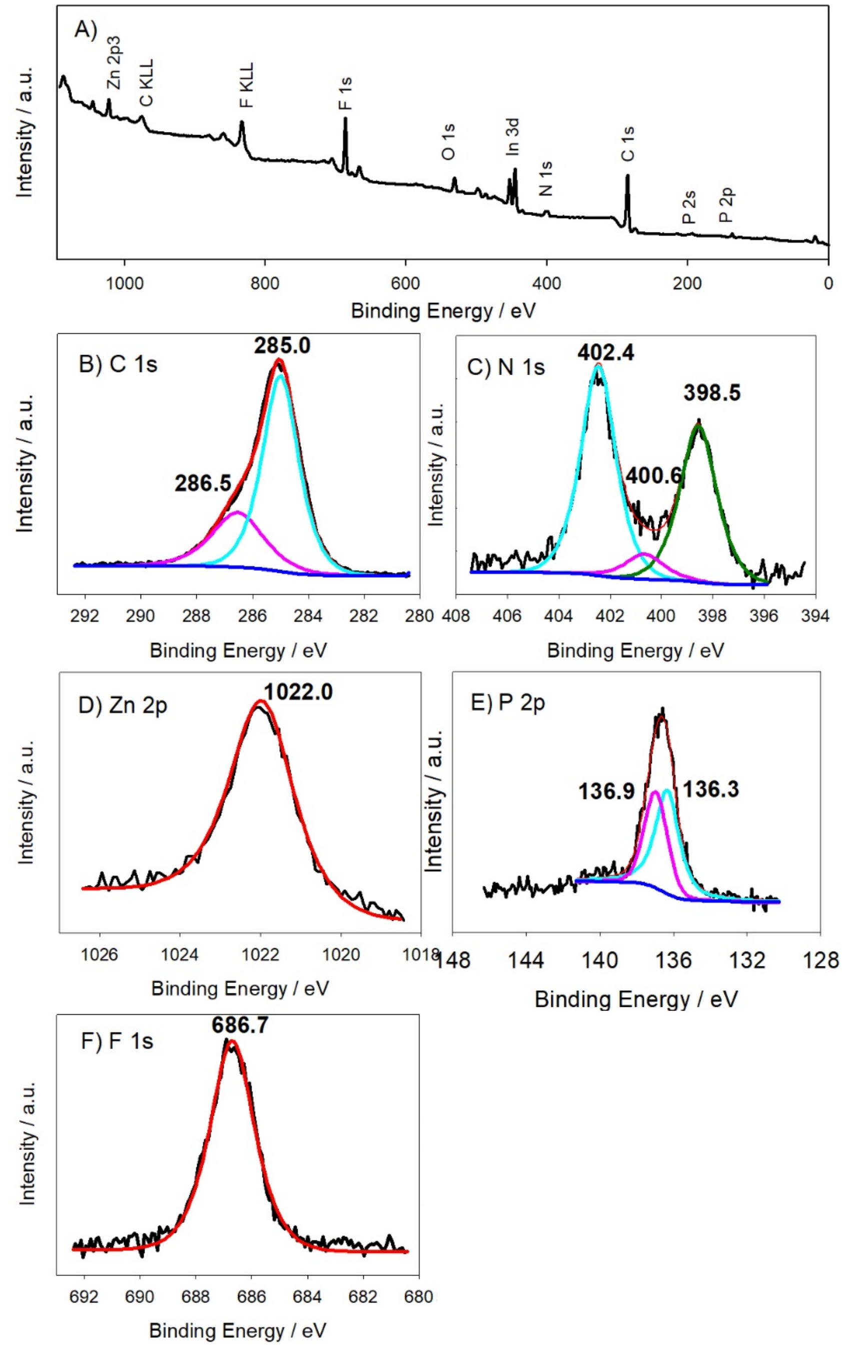

Fig. S3 XPS spectra of the modified ITO electrodes with **poly-ZnT$_2$isoP2** obtained with 1,2-bis(diphenylphosphino)ethene after 25 iterative scans between -1.1 V and 1.0 V *versus* SCE. XPS full spectra (A), C 1s (B), N 1s (C), Zn 2p (D), P 2p (E), F 1s (F).

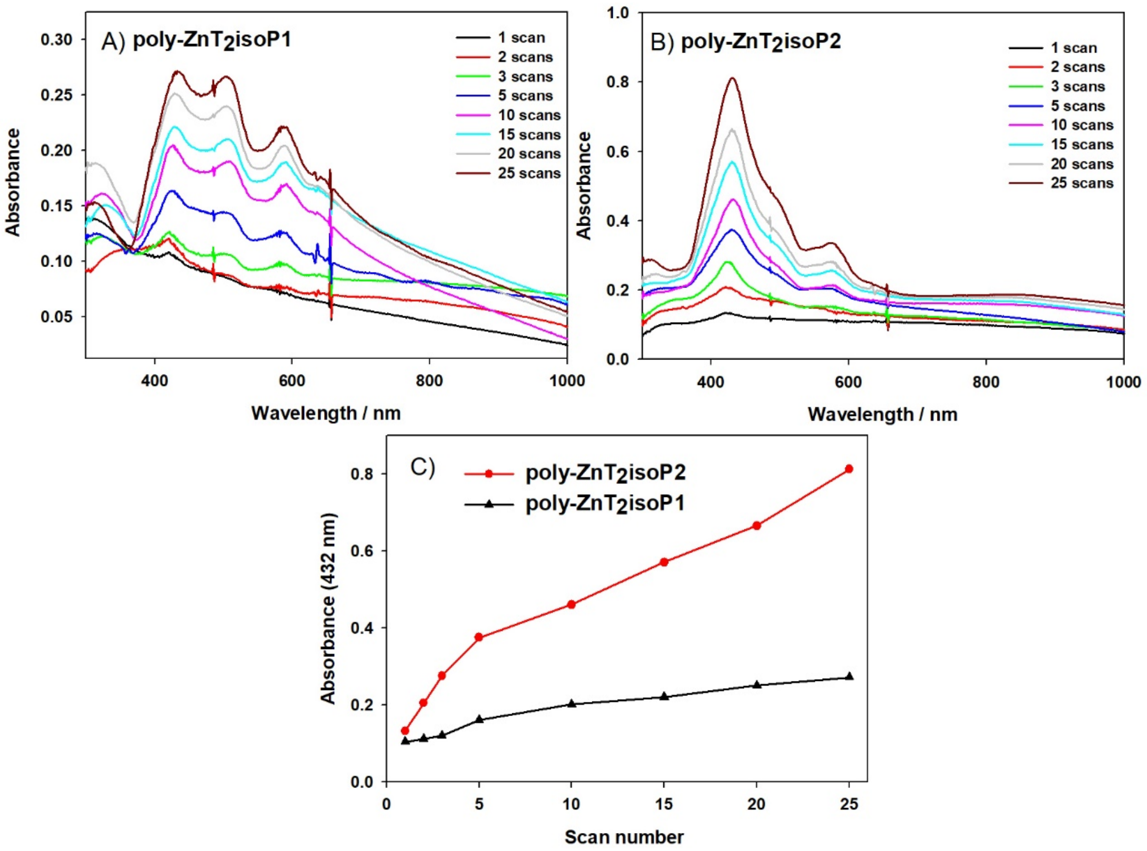

Fig. S4 UV–vis spectra of A) **poly-ZnT$_2$isoP$_1$** and B) **poly-ZnT$_2$isoP$_2$** obtained after n iterative scans (n = 1, 2, 3, 5, 10, 15, 20 and 25) on ITO. C) Plot of the intensity of the absorbance of the Soret band (432 nm) *versus* the iterative scan number between -1.1 V and +1.00 V *versus* SCE.

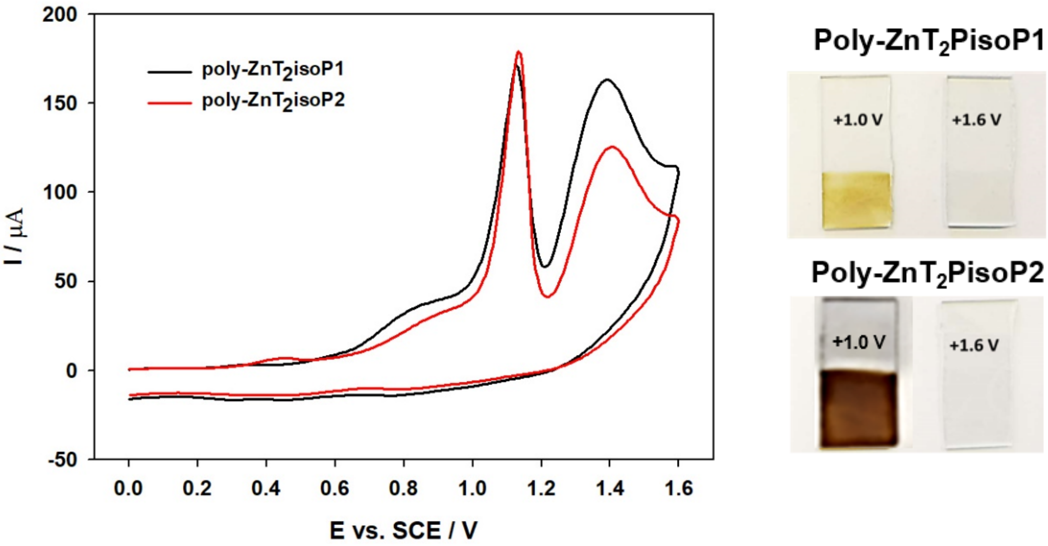

Fig. S5 Cyclic voltammograms of **poly-ZnT$_2$isoP1** and **poly-ZnT$_2$isoP2** obtained after n = 10 scans using iterative scan between -1.1 V and +1.0 V *vs.* SCE in CH_3CN/1,2-$C_2H_4Cl_2$ (3/7) with 0.1 mol.L^{-1} TBAPF$_6$. WE: ITO. S = 1 cm^2. v = 0.1 V.s^{-1}. Right: Pictures of **poly-ZnT$_2$isoP1** and **poly-ZnT$_2$isoP2** copolymer films deposited at 25 scans between -1.1 and 1.0 V *vs.* SCE on the ITO and after oxidization to applied potential of 1.6 V *vs.* SCE on the ITO.

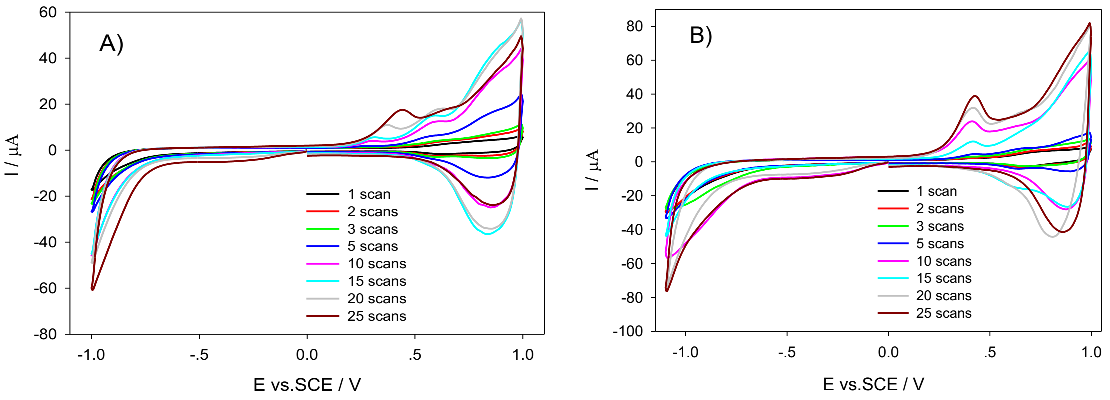

Fig. S6 Cyclic voltammograms of (A) **poly-ZnT$_2$isoP1** between-1.0 V and +1.0 V and (B) **poly-ZnT$_2$isoP2** after n = 1, 3, 5, 10, 15 and 20 scans between-1.1 V and +0.9 V in $CH_3CN/1,2\text{-}C_2H_4Cl_2$ (3/7) with 0.1 M NBu_4PF_6. WE: ITO. S = 1 cm^2, v = 0.1 V s^{-1}. Irreversible peaks not labelled in anodic part correspond to the oxidation of the π-ring of the macrocycle.

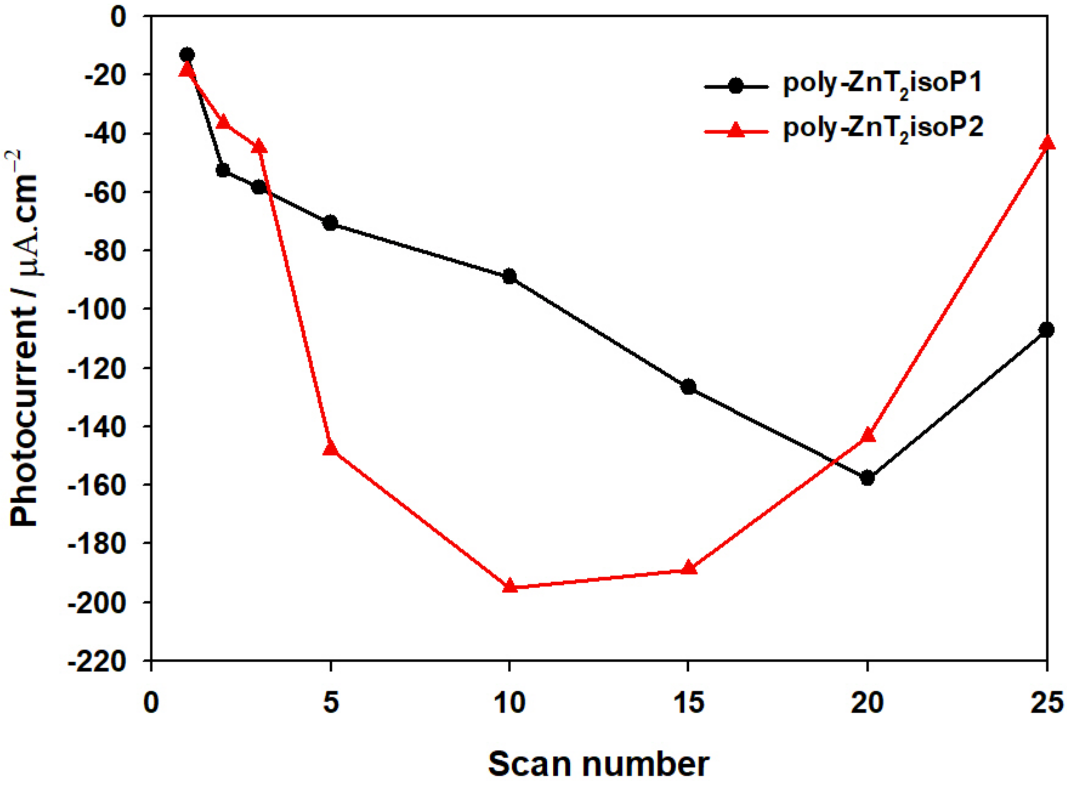

Fig. S7 Photoelectrochemical response of **poly-ZnT$_2$isoP1** (black line) and of **poly-ZnT$_2$isoP2** (red line) obtained with n = 1, 2, 3, 5, 10, 15, 20 and 25 iterative scans.

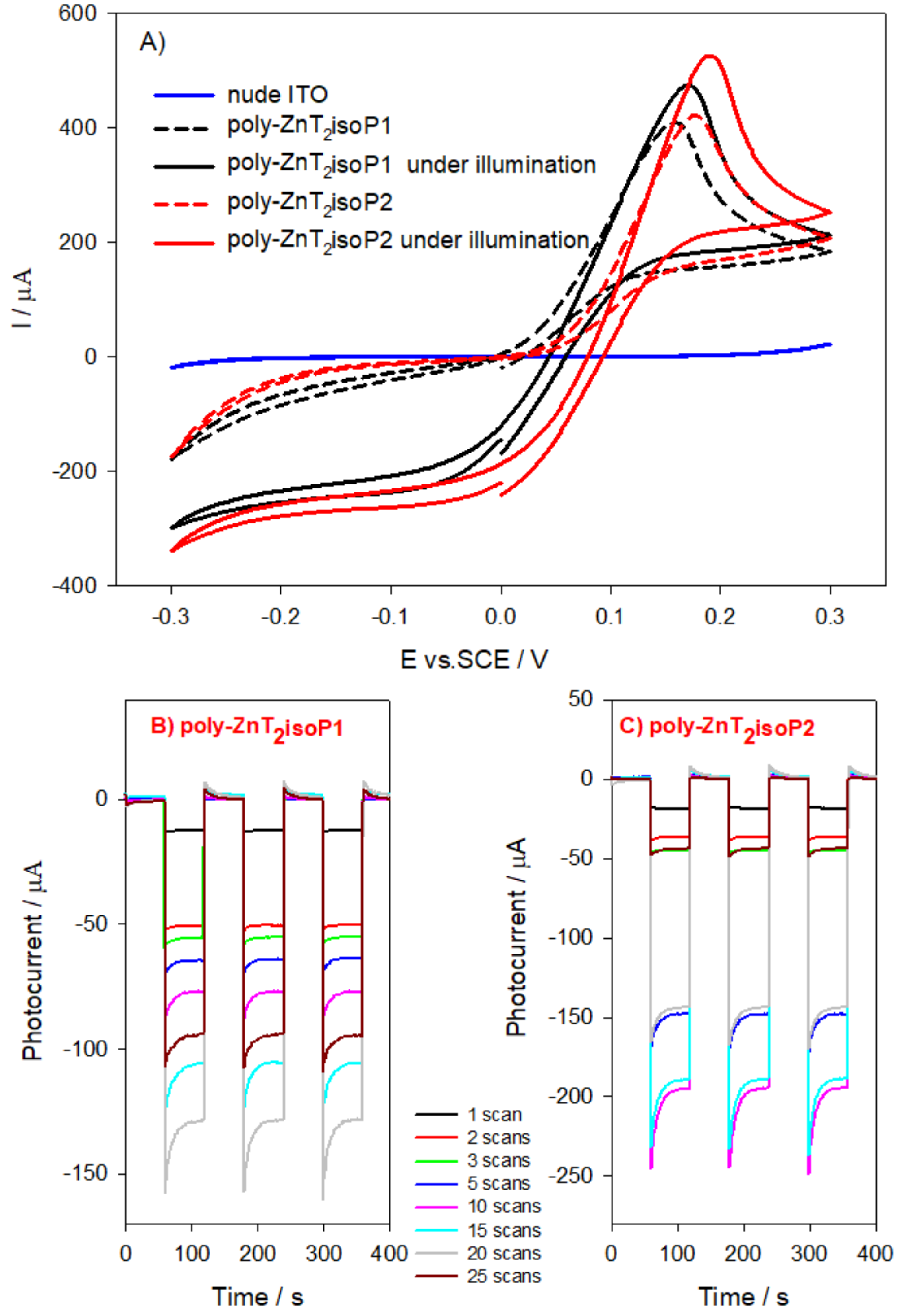

Fig. S8 A) Current-potential curves of **poly-ZnT₂isoP1** (obtained with n = 20 iterative scans between -1.0 V and +1.0 V *vs.* SCE) and **poly-ZnT₂isoP2** (obtained with n = 10 iterative scans between -1.1 V and +1.0 V *vs.* SCE) thin films on ITO electrodes in 0.5 M I^- / 5 mM I_3^- aqueous solution in the dark or under visible illumination. B) and C) Photoelectrochemical response of **poly-ZnT₂isoP1** and **poly-ZnT₂isoP2** films obtained with n = 1, 2, 3, 5, 10, 15, 20 or 25 iterative scans. Measurements has been done under on-off light illumination from a 300 W Xe arc lamp (with λ > 385 nm long pass filter) in I_3^- 5 mmol L^{-1} and I^- 0.5 mol L^{-1} aqueous solution. BIAS potential: 0.00 V *vs.* OCP.

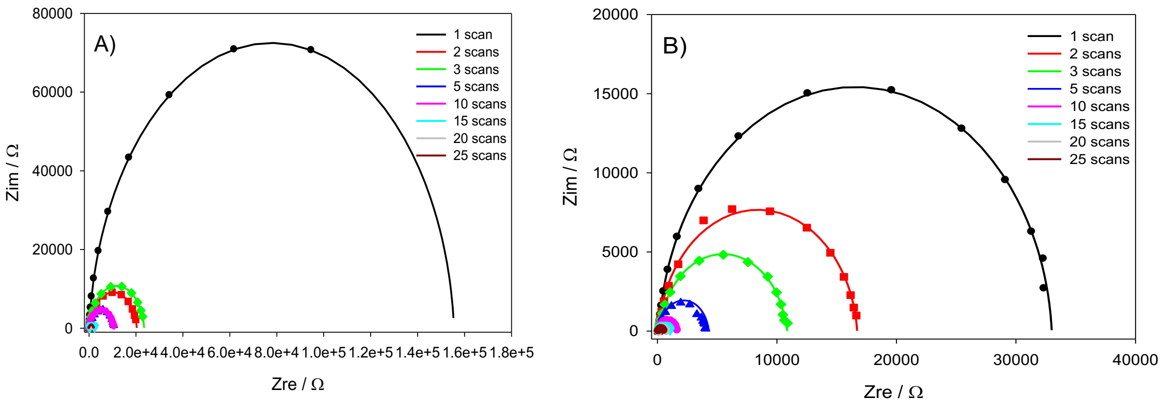

Fig. S9 A) ESI Nyquist of **poly-ZnT$_2$isoP1** and B) **poly-ZnT$_2$isoP2**. Measurements has been done in the dark and in H_2O containing I_3^- 5 mmol.L^{-1} and I^- 0.5 mol.L^{-1}. BIAS potential: 0.00 V *vs.* OCP.

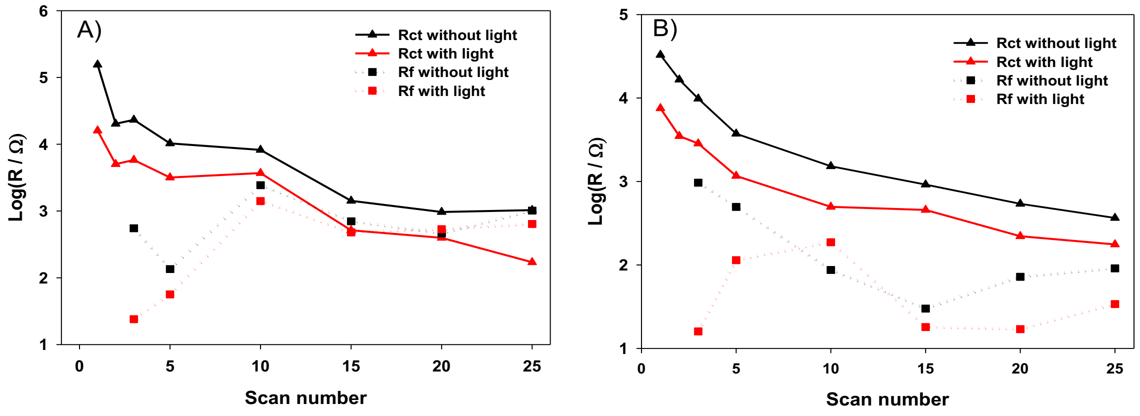

Fig. S10 Plot of the Log(R) *versus* n (number of iterative scan, v = 100 mVs^{-1}) for (A) **poly-ZnT$_2$isoP1** and (B) **poly-ZnT$_2$isoP2**. Measurements has been done in H_2O under on (red line) and off light (black line) illumination from a 300W Xe arc lamp (with λ > 385 nm long pass filter) in containing I_3^- 5 mmol L^{-1} and I^- 0.5 mol L^{-1}. BIAS potential: 0.00 V *vs.* OCP. (R_{ct} = charge transfer resistance at the ITO/copolymer interface (triangle plot with solid line), R_f = the charge transfer resistance of films (square plot with dotted line).

Table S1. Mass change calculated from CV and Faraday's law compared with the masse change measured from EQCM during CV measurements during electropolymerization for **poly-ZnT$_2$isoP1** and **poly-ZnT$_2$isoP2**.

Repeat unit	Molecular Mass per repeat unit (g/mol)	Δm_{calc} / Δm_{EQCM} 3 scans (µg/cm^2)	Δm_{calc} / Δm_{EQCM} 5 scans (µg/cm^2)	Δm_{calc} / Δm_{EQCM} 10 scans (µg/cm^2)	Δm_{calc} / Δm_{EQCM} 20 scans (µg/cm^2)
**poly-ZnT$_2$isoP1** Repeat unit: isoZnT$_2$P-$^+$PPh$_2$-HC=CH-PPh$_2^+$ • 2PF$_6^-$	1236	1.18 / 0.77	1.86 / 1.74	3.54 / 3.69	7.26 / 6.66
**poly-ZnT$_2$isoP2** Repeat unit: isoZnT$_2$P-$^+$PPh$_2$-Ph-PPh$_2^+$ • 2PF$_6^-$	1286	1.72 / 1.76	2.70 / 3.15	5.00 / 5.82	9.49 / 9.18

Δm_{EQCM} is the mass deposited measured from EQCM measurements during electropolymerization.

Δm_{calc} = MQ/(nFA).

M is the molecular mass of repeat units (g/mol)

Q is the charge (C) calculated from the reduction waves of CV of copolymer films obtained from -1.00 V to 1.0 V *vs.* SCE.

n = electron number.

F = Faraday's constant 96485 C/mol.

A = electrode surface (1 cm^2).
